# Clinical and economic research of bone modifiers as adjuvant therapy for early breast cancer: A systematic literature review

**DOI:** 10.1016/j.breast.2025.104551

**Published:** 2025-08-07

**Authors:** Wenhua Wu, Yixiao Zhu, Huiting Lin, Jia Liu, Suyan Liu, Lirong Zhang, Jiaqin Cai, Hong Sun, Xiaoxia Wei

**Affiliations:** a Department of Pharmacy, Fujian Provincial Hospital, Shengli Clinical Medical College of Fujian Medical University, Fuzhou University Affiliated Provincial Hospital, Fuzhou 350001, PR China; b School of Pharmacy, Fujian Medical University, Fuzhou, Fujian 350001, PR China; c School of Pharmacy, Fujian University of Traditional Chinese Medicine, Fuzhou, Fujian 350001, PR China

**Keywords:** Bone modifiers, Bisphosphonates, Denosumab, Breast cancer, Adjuvant therapy

## Abstract

**Background:**

Adjuvant bisphosphonate therapy is recommended by some guidelines for postmenopausal breast cancer, but its application is not ideal due to inconsistent study results and economic burden considerations. This review aims to provide a comprehensive overview of bone modifiers in breast cancer adjuvant treatment to inform clinical practice and health policy.

**Methods:**

PubMed, Embase, Cochrane Library, and Web of Science were searched using terms related to bone modifiers and breast cancer. The included studies comprised clinical trials evaluating adjuvant bone modifiers in early breast cancer (EBC) that reported recurrence, metastasis, or survival outcomes, as well as economic studies that reported costs and effects. Quality was assessed using Cochrane Risk of Bias tool and Quality of Health Economic Studies scale. We also summarized current international guideline recommendations regarding adjuvant bone modifiers in EBC.

**Results:**

Of the 31 eligible articles, findings showed that zoledronic acid and clodronate demonstrated reduced recurrence and improved survival in low-estrogen EBC patients, whereas ibandronate showed no significant benefit. Other bisphosphonates and denosumab require further investigation. Bone modifiers were generally well-tolerated, with mild adverse events. Serious events like nephrotoxicity, osteonecrosis of the jaw, and atypical femoral fractures were rare but necessitate monitoring and prevention. Economic studies suggest that adjuvant zoledronic acid may be cost-effective for postmenopausal EBC patients.

**Conclusion:**

Many unresolved issues remain regarding bone modifiers in EBC adjuvant therapy. Insufficient clinical and economic evidence precludes drawing comprehensive conclusions at present. Future studies need to provide higher-quality evidence to deepen our understanding of adjuvant therapy with bone modifiers.

## Highlights

• Systematic review of bone modifiers in early breast cancer adjuvant therapy.
• Zoledronic acid and clodronate show potential benefits in reducing recurrence risk and improving survival.
• Ibandronate does not demonstrate significant advantages for adjuvant use and denosumab warrants further investigation.
• Economic analysis supports zoledronic acid's cost-effectiveness in certain contexts.
• Further research needed to clarify bone modifiers' role in adjuvant therapy.

## Graphical abstract

**Figure:**
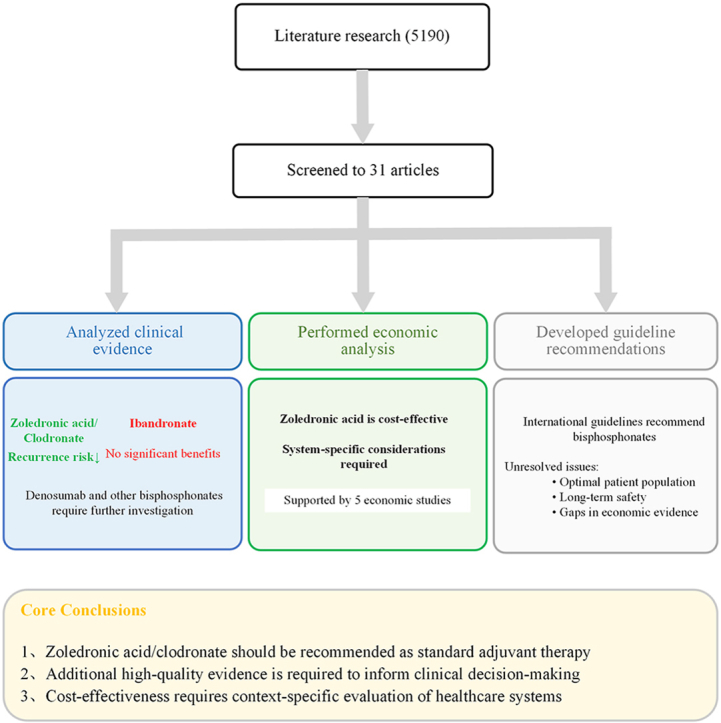

## 1 Introduction

Breast cancer is the leading malignancy in women, with 2.3 million women diagnosed annually and 685,000 deaths from breast cancer-related diseases worldwide [1]. Of new breast cancer cases, 98.3 % are early breast cancer (EBC). Although the long-term prognosis for EBC patients has improved, recurrence and metastasis remain major treatment challenges. 20–30 % of breast cancer patients may develop metastases after diagnosis and treatment of the primary tumor, and metastases account for approximately 90 % of cancer-related deaths [2]. Bone is the most common site of breast cancer recurrence, accounting for approximately 75 % of metastatic cases [3], with a 5-year overall survival rate of 22.8 % [4].

Bone modifiers, including bisphosphonates and denosumab, are established as therapeutic agents for tumor bone metastases, hypercalcemia, and osteoporosis [5]. Recently, bisphosphonates and denosumab have attracted attention due to their potential to reduce disease recurrence and metastasis in the adjuvant treatment of EBC. However, clinical trials assessing various bisphosphonates and denosumab as adjuvant treatments for EBC have reported inconsistent outcomes. The Early Breast Cancer Trialists' Collaborative Group (EBCTCG) pooled analysis of individual patient data showed a modest reduction in bone recurrence (rate ratio [RR] 0.83, p = 0.004) and breast cancer-specific mortality (RR 0.91, p = 0.04) among EBC patients treated with adjuvant bisphosphonate. This benefit was limited to postmenopausal women, including natural or induced menopause (either potentially reversibly via gonadotropin-releasing hormone [GnRH] analogues or permanently by oophorectomy), without impacting local or extraosseous recurrence [6].

The EBCTCG study has informed international guidelines such as those from the American Society of Clinical Oncology (ASCO) and the European Society of Medical Oncology (ESMO), which recommend considering adjuvant bisphosphonate therapy for postmenopausal women regardless of hormone receptor status, with individualized decisions according to the risk of relapse [7,8]. Nevertheless, concerns have been raised regarding the uncertain efficacy of these treatments, the feasibility of administering repeated infusions, potential toxicity, and the added economic burden [9,10]. At the 2019 St. Gallen Consensus Conference, a discrepancy was evident: while 83.7 % of the panel strongly supported the use of adjuvant bisphosphonate, only 42.6 % reported routine use in practice [11].

In this review, we examined the latest clinical research on the use of bisphosphonates and denosumab in the adjuvant treatment of EBC patients and explored their impact on patient prognosis. We also conducted a detailed analysis of relevant economic studies to assess the acceptability of these treatments from a health economics perspective. Furthermore, we summarized the recommendations from current international guidelines regarding the adjuvant use of bone modifiers. Based on four dimensions—efficacy, safety, economy, and guideline recommendations—this review provides a thorough overview of the use of bone modifiers in the adjuvant treatment of EBC, aiming to provide references for clinical decision-making and a basis for health policy development.

## 2 Methods

This review was conducted according to the Cochrane recommendations [12] and reported following the Preferred Reporting Items for Systematic Reviews and Meta-Analyses (PRISMA) guidelines [13]. The study was registered in the International Prospective Register of Systematic Reviews (PROSPERO, ID CRD42024502493).

### 2.1 Search strategy

We searched PubMed, Embase, Cochrane Library, and Web of Science to identify clinical trials and economic studies on adjuvant bisphosphonates or denosumab for EBC patients, from database inception to June 5, 2024. We also examined the bibliographies of previous reviews and manually searched the cited references for further relevant literature. Search terms included “breast cancer”, “bisphosphonate”, “zoledronate”, “clodronate”, “ibandronate”, “pamidronate”, “denosumab”, “clinical trial”, “randomized controlled trial”, “cost-effectiveness analysis”, “cost-utility analysis”, etc. To ensure a comprehensive retrieval of papers, we utilized both MeSH and free-text terms with English language. The complete search strategy is shown in Supplementary Table 1. Two authors independently assessed the articles for eligibility, with disagreements resolved through discussion or third-party consultation.

### 2.2 Inclusion and exclusion criteria

In this literature review, the included clinical trials evaluated adjuvant bisphosphonates or denosumab against certain controls (no treatment, placebo, other bisphosphonates, or different administrations of the same bisphosphonate). Trials designed to measure breast cancer recurrence, metastasis, or survival provided the most powerful evidence. Trials solely on bone effects, like bone mineral density (BMD), were excluded unless they also detailed recurrence or survival outcomes.

Additionally, the included economic studies were required to report both costs and life-years or quality-adjusted life years (QALYs) saved by adjuvant bone modifiers in EBC patients. Studies reporting only costs or effects, or those assessing the economics of bone modifiers in bone density loss treatment or prevention, were excluded.

### 2.3 Quality assessment

We assessed the risk of bias in randomized controlled trials (RCTs) using the Cochrane Collaboration Risk of Bias Tool (Cochrane ROB) [14], deeming the risk low if all domains were judged to be at low risk, and unclear or high if any domain was judged to be unclear or at high risk. We also assessed the quality of each economic study using the Quality of Health Economic Studies Instrument (QHES), which consists of 16 yes/no questions [15,16]. Each question scores 1 to 9 points, with 'yes' answers earning full credit and ‘no’ answers scoring zero. The total score ranges from 0 to 100 (from poorest to highest), with 0–24 indicating extremely poor quality, 25–49 poor quality, 50–74 fair quality, and 75–100 high quality.

## 3 Results

**Fig. 1:**
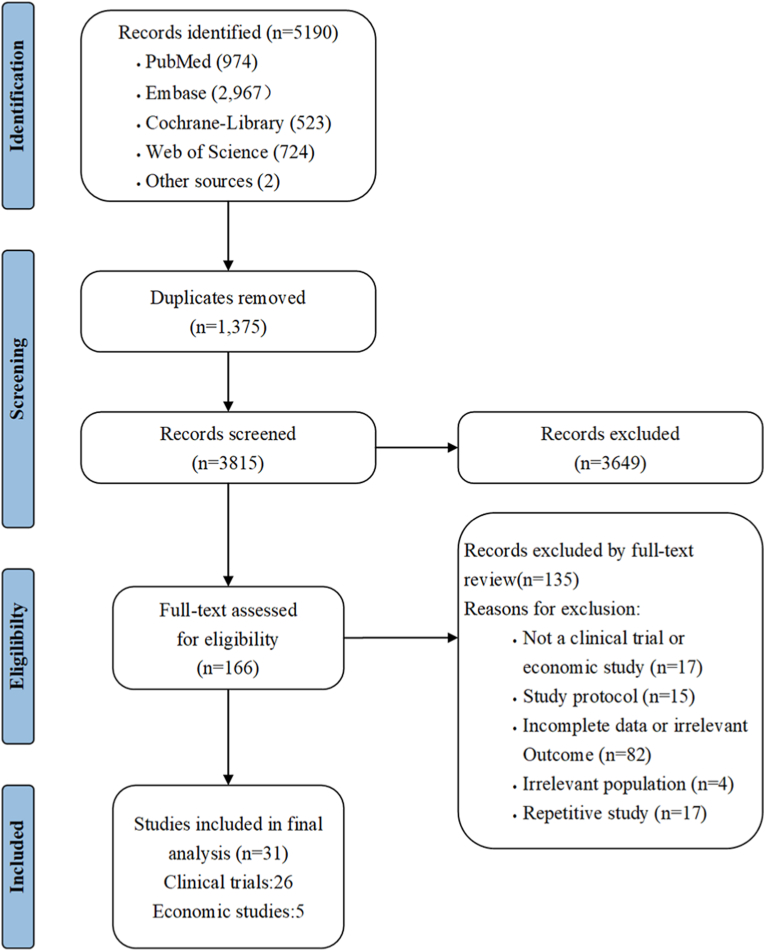
Flowchart of the search and selection process for studies identified in the systematic review. “n” represents the number of studies.

Our literature search yielded 5190 citations, from which 532 articles were retained after excluding duplicates and ineligible studies. As shown in Fig. 1, 26 articles were ultimately included, reporting 22 clinical trials with a total of 36,579 participants. These trials evaluated five bone modifies: zoledronic acid, clodronate, ibandronate, pamidronate, and denosumab. Table 1 summarizes the baseline characteristics of the study populations, including tumor stage, menopausal status, hormone receptor status, and median follow-up time, etc. Table 2 presents key efficacy and safety outcomes, such as disease-free survival (DFS), overall survival (OS), incidence of bone metastases, adverse events (AEs), grade ≥3 AEs, osteonecrosis of the jaw. Of the 22 trials, 21 were randomized controlled trials (RCT) [[17], [18], [19], [20], [21], [22], [23], [24], [25], [26], [27], [28], [29], [30], [31], [32], [33], [34], [35], [36], [37], [38], [39], [40], [41]] and one was a non-RCT [42].

**Table 1:** Baseline characteristics of clinical trials evaluating adjuvant bone modifiers in early breast cancer.

Trial	Design	Sample Size	Tumor Stage	Menopausal Status	Hormone Receptor Status	Bone modifiers schedule	Median age (years)	Median Follow-up time (months)	Primary endpoint	Secondary endpoint
AZURE (BIG 01/04) [17,18]	RCT, phase 3	3359	Stage II/III	Premenopausal: 1504 (44.8 %) Perimenopausal: 490 (14.6 %) Postmenopausal: 1041 (31.0 %) Unknown: 324 (9.6 %)	ER+: 2633 (78.4 %) ER−: 706 (21.0 %) Unknown: 20 (0.6 %) PR+: 1424 (42.4 %) PR−: 806 (24.0 %) Unknown: 1129 (33.6 %)	ZA: 4 mg IV every 3–4 weeks × 6, then 3-monthly × 8, 6-monthly × 5 (total 5 years)	51.5	117	DFS	IDFS, OS, safety, TTBM, skeletal morbidity
Leal 2010 [19]	RCT	68	Stage II/III	Postmenopausal: 68 (100 %)	ER/PR+: 58 (85 %) ER/PR-: 10 (15 %)	ZA: 4 mg IV every 3 months for 4 cycles	ZA group: 54.5 (41–83); No-treatment group: 50.5 (37–65)	96	BMD (12 months)	BMD, DFS, OS, toxicity
ABCSG-12 [20]	RCT, phase 3	1803	Stage I/II	Premenopausal women received goserelin: 1803 (100 %)	HR+: 1803 (100 %)	ZA: 4 mg every 6 months for 3 years	45.0	94.4	DFS	Recurrence-free survival, OS, safety
HOBAE [21]	RCT, phase 3	1065	Stage I/II	Premenopausal women received triptorelin: 1065 (100 %)	HR+: 1065 (100 %)	ZA: 4 mg IV every 6 months for 5 years	45.0	100.4	DFS	OS, toxicity
Banys 2013 [22]	RCT, phase 2	86	Stage I/II	Premenopausal: 55 (64 %) Postmenopausal: 31 (36 %)	ER+: 60 (69.8 %) ER-: 9 (10.5 %) PR+: 47 (54.7 %) PR-: 22 (25.6 %)	ZA: 4 mg IV every 4 weeks for 24 months	–	88	DTC (12 months)	DTC (24 months), safety, BMFS, OS
NaTaN [23]	RCT, phase 3	693	Stage II/III	Premenopausal: 185/693 (27.5 %); Postmenopausal: 316/693 (47.0 %); Missing: 21/693 (3.0 %)	ER/PR-: 143 (20.7 %)ER/PR+: 548 (79.3 %)	ZA: 4 mg IV every 4 weeks for 6 months, then every 3 months for 2 years, then every 6 months for 2.5 years (total 19 infusions)	≤55: 66.4 % >55: 33.6 %	54.7	DFS	OS, BMFS, toxicity
E-ZO-FAST [24,25]	RCT, phase 3	527	Stage I to IIIa	Established Postmenopausal: 438 (83.9 %) Recently Postmenopausal: 84 (16.1 %)	HR+: 100 %	Immediate ZA: 4 mg IV every 6 months for 5 years beginning on Day 1 Delayed ZA: 4 mg IV every 6 months, started when BMD T-score < −2.0 or fracture	58.0	12	BMD (12 months)	total hip BMD, fracture incidence, disease recurrence, safety
Z-FAST [26]	RCT, phase 3	600	Stage I-IIIA	Postmenopausal: 600 (100 %)	HR+: 600 (100 %)	Immediate ZA: 4 mg IV every 6 months for 5 years beginning on Day 1 Delayed ZA: 4 mg IV every 6 months, started when BMD T-score < −2.0 or fracture	60.0	61	BMD (12 months)	BMD (24, 36, 48, 61 months), fracture incidence; disease recurrence; safety
ZO-FAST [27]	RCT, phase 3	1065	Stage I-IIIA	Recently postmenopausal: 177 (16.6 %) Truly postmenopausal: 888 (83.4 %)	ER+ and/or PR+: 1065 (100 %)	Immediate ZA: 4 mg IV every 6 months for 5 years for 5 years beginning on Day 1 Delayed ZA: 4 mg IV every 6 months, initiated when BMD T-score < −2.0 or fracture occurred	57.5	60	BMD (12 months)	BMD (24, 36, 48, 60 months), DFS, OS, safety
SUCCESS A [28]	RCT, phase 3	2987	Stage II/III	Premenopausal: 1263 (42.3 %) Postmenopausal: 1724 (57.7 %)	HR+: 2226 (74.5 %) HR-: 406 (13.6 %) Unknown: 355 (11.9 %)	5-year ZA: 4 mg IV every 3 months for 2 years, then every 6 months for 3 years 2-year ZA: 4 mg IV every 3 months for 2 years	53.0	35.4	DFS	OS, DDFS, skeletal-related adverse events
Saarto 2004 [29]	RCT	282	Stage II/III	Premenopausal: 148 (52.5 %) Postmenopausal: 134 (47.5 %)	ER+: 182 (64.5 %) ER-: 81 (28.7 %) ER Unknown: 19 (6.8 %) PR+: 156 (55.3 %) PR-: 106 (37.6 %) PR Unknown: 20 (7.1 %)	Clodronate: Oral clodronate 1600 mg daily for 3 years	52.0	120	DFS	OS, bone metastases, non-skeletal metastases
Diel 2008 [30]	RCT	302	Stage I–IIIA	Postmenopausal: 189 (63 %) Premenopausal: 113 (37 %)	ER+: 188 (75 %) ER-: 63 (25 %) PR+: 157 (62 %) PR-: 96 (38 %)	Clodronate: Oral clodronate 1600 mg/day for 2 years	51.0	103	OS	DFS, bone metastases, visceral metastases
NSABP B-34 [31]	RCT, phase 3	3323	Stage I–III	Premenopausal: 1177 (35.5 %) Postmenopausal: 2134 (64.5 %)	HR+: 2586 (78 %) HR-: 736 (22 %)	Clodronate: Oral clodronate 1600 mg daily for 3 years	55.0	90.7	DFS	OS, recurrence-free interval, bone metastasis-free interval, non-bone metastasis-free interval
Powles 2006 [32]	RCT	1069	Stage I–III	Premenopausal: 530 (50 %) Postmenopausal: 539 (50 %)	ER+: 485 (45.4 %) ER-: 272 (25.4 %) ER Unknown: 312 (29.2 %) PR+: 228 (21.3 %) PR-: 154 (14.4 %) PR Unknown: 687 (64.3 %)	Clodronate: Oral clodronate 1600 mg/day for 2 years	clodronate group: 52.8 placebo group: 52.7	67.2	Time to first bone metastases over 5 years	Overall survival, occurrence of non-skeletal relapses
GAIN [33]	RCT, phase 3	2994	Stage II/III	Premenopausal: 1622 (54.2 %) Postmenopausal: 1372 (45.8 %)	HR+: 2171 (72.5 %) HR-: 823 (27.5 %)	Ibandronate: Oral ibandronate 50 mg/day for 2 years	ibandronate group: 49.0 observation group: 50.0	38.7	DFS	OS, safety, subgroup event-free survival
TEAM-IIB [34]	RCT, phase 3	1116	Stage I–III	Postmenopausal: 1116 (100 %)	ER+/PR+: 823 (73.7 %) ER+/PR-: 287 (25.7 %) ER-/PR+: 6 (0.5 %)	Ibandronate: Oral ibandronate 50 mg daily for 3 years	62.0	102	DFS	OS, recurrence-free interval, BMFS, safety
BONADIUV [35]	RCT, phase 2	171	stage I–III	Postmenopausal: 171 (100 %)	HR+: 171 (100 %)	Ibandronate: Oral ibandronate 150 mg monthly for 2 years	60.2	63.3	BMD	AEs, iDFS, OS, bone metastases
S0307 [36]	RCT, phase 3	6097	stage I–III	Postmenopausal: 6097 (100 %)	ER+/PR+: 4725 (78.5 %) ER-/PR-: 1286 (21.4 %)	ZA: IV 4 mg monthly for 6 months, then every 3 months for 2.5 years Clodronate: oral 1600 mg daily for 3 years Ibandronate: oral 50 mg daily for 3 years	52.7	60.00	DFS	OS, site of first recurrence, toxicity
Kristensen 2008 [37]	RCT, phase 3	953	stage I–II	Premenopausal: 634 (66.5 %) Postmenopausal: 318 (33.4 %) Unknown: 1 (0.1 %)	ER+: 147 (15.4 %) ER-: 539 (56.5 %) ER unknown: 267 (28.0 %) PR+: 105 (11.0 %) PR-: 271 (28.4 %) PR unknown: 577 (60.5 %)	Pamidronate: Oral pamidronate 150 mg twice daily for 4 years	49.0	120	Occurrence of bone metastases	BMD
Kokufu 2010 [42]	Non-RCT	90	Stage II/III	Premenopausal: 40 (44.4 %) Postmenopausal: 50 (55.6 %)	ER+: 37 (41.1 %) ER-: 50 (55.6 %) ER unknown: 3 (3.3 %) PR+: 32 (35.6 %) PR-: 55 (61.1 %) PR unknown: 3 (3.3 %)	Pamidronate: Intravenous pamidronate 45 mg every 2 weeks for 4 infusions	Pamidronate group: 54.0 No-treatment group: 50.0	Pamidronate: 61.1 (29.5–71.6); No-treatment: 82.9 (30.9–99.8)	Incidence of bone metastases	Distant metastases, non-osseous metastases, OS, DFS
ABCSG-18 [38,39]	RCT, phase 3	3420	Stage I–III	Postmenopausal: 3420 (100 %)	ER+: 3384 (99 %) PR+: 2874 (84 %) ER-: 36 (1 %) PR+: 543 (16 %)	Denosumab: Subcutaneous denosumab 60 mg every 6 months	64.0	73	Time to first clinical fracture	DFS, BMD, OS, vertebral fractures
D-CARE [40,41]	RCT, phase 3	4509	Stage II/III	Premenopausal: 2360 (52.3 %) Postmenopausal: 2149 (47.7 %)	HR+: 3492 (77.5 %) HR-: 1015 (22.5 %)	Denosumab: Subcutaneous denosumab 120 mg every 3–4 weeks for 6 months, then every 3 months for 4.5 years	51	67.2	BMFS	DFS, OS, time to bone metastasis, fractures, skeletal-related events

AE represents adverse event; BMD represents bone mineral density; BMFS represents bone metastasis-free survival; DFS represents disease-free survival; DTC represents disseminated tumor cells; ER represents estrogen receptor; HR represents hormone receptor; IDFS and iDFS represent invasive disease-free survival; IV represents intravenous; OS represents overall survival; PgR/PR represents progesterone receptor; RCT represents randomized controlled trial; and TTBM represents time to bone metastasis.

**Table 2:** Efficacy and safety outcomes of adjuvant bone modifiers in early breast cancer.

Trial	Treatment	Sample	Efficacy					Safety			
			Disease recurrences	Bone metastases	BMFS	DFS	OS	AEs	≥3 grade AEs	ONJ	Nephrotoxicity
AZURE (BIG 01/04) [17,18]	ZA	1681	502 (30 %)	299/1681: 352/1678, HR 0.76 (95 %CI 0.63–0.92, p = 0.005)	–	555/1681: 575/1678, HR 0.94 (95 %CI: 0.84–1.06, p = 0.340)	HR 0.92, (95 %CI 0.81–1.05, P = 0.24)	–	–	30 (1.8 %)	32 (1.9 %)
	No-treatment	1678	508 (30 %)		–			–	–	0	12 (0.7 %)
Leal 2010 [19]	ZA	36	–	–	–	–	10 deaths in total (15 %)	–	–	–	–
	No-treatment	32	–	–	–	–		–	–	–	–
ABCSG-12 [20]	ZA	900	111 (11.2 %)	27/900: 35/903, HR 0.76 (95 % CI 0.46–1.25)	–	104/900: 135/903, HR 0.77 (95 % CI 0.60–0.99, p = 0.042)	HR 0.66 (95 % CI 0.43–1.02, p = 0.064)	700 (77.8 %)	–	0	0
	No-treatment	903	140 (15.6 %)		–			657 (72.6 %)	–	0	0
HOBAE [21]	Tamoxifen(T)	354	84 (23.7 %)	17 (4.8 %)	–	ZL vs L: HR 0.70 (95 % CI, 0.44–1.12, p = 0.22) ZL vs T: HR 0.52 (95 % CI, 0.34–0.80, p = 0.003) L vs T: HR 0.72 (95 % CI, 95 % CI, 0.48–1.07, p = 0.06	34 deaths (9.6 %)	–	16 (4.6 %)	0	–
	Letrozole(L)	356	60 (16.9 %)	11 (3.1 %)	–		25 deaths (7.0 %)	–	29 (8.0 %)	0	–
	ZA + letrozole (ZL)	355	55 (15.5 %)	13 (3.7 %)	–		20 deaths (5.6 %)	–	31 (9.5 %)	6 (1.8 %)	–
Banys 2013 [22]	ZA	40	3 (8 %)	–	–	–	1/40: 5/46, HR 0.18 (95 % CI: 0.02–1.45, p = 0.11)	–	–	0	–
	No-treatment	46	7 (15 %)	–	–	–		–	–	0	–
NaTaN [23]	ZA	343	–	65 (19.0 %)	HR 1.08, 95 % CI 0.76–1.54, P = 0.658	82/343: 87/350, HR 0.960 (95 % CI 0.709–1.30)	HR 1.19 (95 % CI 0.79–1.79)	197 (86.4 %)	67 (29.0 %)	5 (1.5 %)	7 (2.0 %)
	No-treatment	350	–	61 (17.4 %)				179 (75.0 %)	49 (21.0 %)	0	4 (1.1 %)
E-ZO-FAST [24,25]	Immediate ZA	252	7 (2.8 %)	3 (1.2 %)	–	–	3 deaths (1.2 %)	221 (87.7 %)	–	2 (0.8 %)	1 (0.4 %)
	Delayed ZA	270	5 (1.9 %)	5 (1.9 %)	–	–	0	242 (89.6 %)	–	0	0
Z-FAST [26]	Immediate ZA	300	16 (5.3 %)	3 (1.0 %)	–	25/300: 23/300, HR 0.91 (95 % CI 0.56–1.48)	4/300: 7/300, HR 1.75 (95 % CI 0.56–5.45)	282 (94.0 %)	38 (12.7 %)	0	6 (2.0 %)
	Delayed ZA	300	21 (7.0 %)	7 (2.3 %)	–			274 (91.3 %)	30 (10.0 %)	0	4 (1.3 %)
ZO-FAST [27]	Immediate ZA	532	37 (7.0 %)	14 (2.6 %)	–	42/532: 62/533, HR = 0.66 (95 % CI 0.44–0.97, p = 0.0375)	HR 0.69 (95 % CI 0.42–1.14, p = 0.1463)	495 (93.0 %)	78 (14.7 %)	3 (0.3 %)	3 (0.6 %)
	Delayed ZA	533	59 (11.1 %)	24 (4.5 %)	–			486 (91.2 %)	68 (12.8 %)	0	2 (0.4 %)
SUCCESS A [28]	5-year ZA	1540	–	28/1447: 25/1540, HR 0.80, (95 % CI 0.47–1.38)	–	129/1540: 121/1447, HR 0.97 (95 % CI 0.75–1.25, P = 0.81)	59/1540: 57/1147, HR 0.98 (95 % CI 0.67–1.42, P = 0.90)	712 (46.2 %)	117 (7.6 %)	11 (0.7 %)	–
	2-year ZA	1447	–		–			394 (27.2 %)	74 (5.1 %)	5 (0.3 %)	–
Saarto 2004 [29]	Clodronate	139	76 (55 %)	44/139: 42/143, HR1.23 (95 % CI 0.80–1.88, p = 0.35)	–	60/139: 76/143, HR 1.52 (95 %CI 1.08–2.13, p = 0.02)	64/139: 55/143, RR 1.33 (95 % CI 0.92–1.91, p = 0.13)	–	–	–	–
	No-treatment	143	60 (42 %)		–			–	–	–	–
Diel 2008 [30]	Clodronate	157	–	37 (23.6 %)	–	61/157: 57/145, HR 0.81 (95 %CI 0.59–1.12)	32/157: 59/145, HR 0.38 (95 %CI 0.23–0.63, p = 0.049)	–	–	0	–
	No-treatment	145	–	38 (26.2 %)	–			–	–	0	–
NSABP B-34 [31]	Clodronate	1662	148 (8.9 %)	61/1655: 80/1656, HR 0.77 (95 % CI 0.55–1.07, p = 0.12)	–	286/1655: 312/1656, HR 0.91 (95 % CI 0.78–1.07, p = 0.27)	140/1655: 167/1656, HR 0.84 (95 % CI 0.67–1.05, p = 0.13)	–	337 (20.9 %)	1 (0.06 %)	4 (0.2 %)
	Placebo	1661	177 (10.7 %)		–			–	345 (21.2 %)	0	0
Powles 2006 [32]	Clodronate	530	139 (26.2 %)	73/539: 51/530, HR 0.69 (95 % CI 0.48–0.99, p = 0.043)	HR 0.77 (95 % CI 0.56–1.08)	172/539: 145/530, HR 0.82 (95 % CI 0.67–1.00)	129/539: 98/530, HR 0.77 (95 % CI 0.59–0.99, p = 0.048)	105 (19.8 %)	12 (2.3 %)	0	–
	Placebo	539	145 (26.9 %)					92 (17.1 %)	8 (1.5 %)	0	–
GAIN [33]	Ibandronate	1996	355 (17.8 %)	78 (3.9 %)	–	270/1996: 135/998, HR 0.945 (95 % CI 0.768–1.161, p = 0.593)	128/1996: 58/998, HR 0.96 (95 % CI 0.71–1.31, p = 0.801)	435 (21.8 %)	105 (5.3 %)	2 (0.1 %)	10 (0.5 %)
	No-treatment	998	188 (18.8 %)	49 (4.9 %)	–			154 (15.4 %)	29 (2.9 %)	0	5 (0.5 %)
TEAM-IIB [34]	Ibandronate	565	77 (13.6 %)	42/565: 51/551, HR 0.83 (95 % CI 0.55–1.25, p = 0.36)	–	128/565: 129/551, HR 0.97 (95 % CI 0.76–1.24, p = 0.81)	87/565: 81/551, HR 1.10 (95 % CI 0.82–1.49, p = 0.52)	473 (83.7 %)	82 (14.5 %)	11 (1.9 %)	7 (1.2 %)
	No-treatment	551	88 (16.0 %)		–			460 (83.5 %)	73 (13.2 %)	1 (0.2 %)	4 (0.7 %)
BONADIUV [35]	Ibandronate	89	10 (11.2 %)	–	–	6/82: 10/89, HR 1.67 (95 % CI 0.69–4.05, P = 0.42)	6/89: 2/82, HR 3.00 (95 % CI 0.64–14.16, P = 0.19)	36 (50.0 %)	7 (9.7 %)	0	0
	Placebo	82	6 (7.3 %)	–	–			39 (54.2 %)	7 (9.7 %)	0	0
S0307 [36]	ZA	2231	286 (12.8 %)	110 (4.9 %)	–	345/2231	238/2231	–	187 (8.8 %)	28 (1.26 %)	3 (0.14 %)
	Clodronate	2235	295 (13.2 %)	108 (4.8 %)	–	376/2235, HR (Clodronate vs ZA) 1.09 (95 % CI 0.94–1.26)	182/2235, HR (Clodronate vs Zoledronic Acid) 0.84 (95 % CI 0.67–1.05)	–	181 (8.3 %)	8 (0.36 %)	1 (0.04 %)
	Ibandronate	1552	209 (13.5 %)	82 (5.3 %)	–	263/1552, HR (Ibandronate vs ZA) 1.06 (95 % CI 0.90–1.24)	154/1552, HR (Ibandronate vs ZA) 0.92 (95 % CI 0.73–1.16)	–	160 (10.5 %)	12 (0.77 %)	7 (0.45 %)
Kristensen 2008 [37]	Pamidronate	460	–	93/450 (20.7 %)	–	–	–	–	–	–	–
	No-treatment	493	–	88/479 (18.4 %)	–	–	–	–	–	–	–
Kokufu 2010 [42]	Pamidronate	33	–	4/33: 23/57, HR 0.20 (95 % CI0.07–0.56, p = 0.005)	HR 1.03 (95 % CI 0.75–1.40)	–	7/33: 17/57	–	–	0	–
	No-treatment	57	–					–	–	0	–
ABCSG-18 [38,39]	Denosumab	1711	240 (14.0 %)	–	HR 0.81 (95 % CI 0.65–1.00)	240/1711: 287/1709, HR 0.82 (95 % CI 0.69–0.98, p = 0.026)	47/1711: 46/1709, HR 0.95 (95 % CI 0.65–1.38)	1367 (80 %)	1339 (79 %)	0	–
	Placebo	1709	287 (16.8 %)	–				1339 (79 %)	515 (30.0 %)	0	–
D-CARE [40,41]	Denosumab	2256	442 (20 %)	155/2256:189/2253, HR 0.82 (95 % CI0.67–1.00, p = 0.06)	292/2256: 305/2253, HR 0.97, 95 % CI 0.82–1.14; p = 0.70	442/2256: 433/2253, HR 1.04 (95 % CI 0.91–1.19)	204/2253: 208/2256, HR 1.03 (95 % CI 0.85–1.25)	2241 (99.3 %)	–	122 (5 %)	–
	Placebo	2253	433 (19 %)					2218 (98.4 %)	–	4 (0.2 %)	–

AE represents adverse event; BMFS represents bone metastasis-free survival; CI represents confidence interval; DFS represents disease-free survival; HR represents hazard ratio; IV represents intravenous; ONJ represents osteonecrosis of the jaw; OS represents overall survival; PR/PgR represents progesterone receptor; RR represents relative risk; T represents tamoxifen; L represents letrozole; ZL represents zoledronic acid plus letrozole; ZA represents zoledronic acid.

Additionally, 5 articles evaluated the economic impact of adjuvant bisphosphonates therapy in EBC patients [[43], [44], [45], [46], [47]], with their characteristics and results presented in Table 3.

**Table 3:** Summary characteristics of the included economic studies.

Study	Methods	Population	Intervention	Incremental Costs (per patient)	Incremental QALY (per patient)	ICER/ICUR	WTP
Delea 2010 [44]	Cost-effectiveness analysis Markov model; discount rate 3 %; 2008 US dollars; US health care system perspective	Premenopausal women with HR-positive early breast cancer	ZA plus ET (goserelin plus tamoxifen or anastrozole) versus ET	• Trial benefit: $4000 • Lifetime benefit:–$2600	• Trial benefit: 0.43 • Lifetime benefit: 1.41	• Trial benefit: $9300/QALY • Lifetime benefit: dominant	$50,000/QALY
Lux 2010 [47]	Cost–utility analysis Markov model; discount rate 3 %; 2009 euros; German healthcare system perspective	Premenopausal women with HR-positive early breast cancer	ZA plus ET (goserelin plus tamoxifen or anastrozole) versus ET	Trial benefit: – €11	Trial benefit: 0.24	Dominant	€5000/QALY
Lamond 2015 [46]	Cost–utility analysis Markov model; discount rate 5 %; 2014 Canadian dollars; Canadian health care system	Women with early-stage endocrine-sensitive breast cancer and low levels of estrogen because of induced or natural menopause	ZA plus ET versus ET	• Induced menopause group: $7825 • Natural menopause group: $7789	• Induced menopause group: 0.46 • Natural menopause group: 0.34	• Induced menopause group: $17,007/QALY • Natural menopause group: $23,093/QALY	$100,000/QALY
National Guideline Alliance (UK), 2018 [43]	Cost-effectiveness analysis Partitioned survival model; discount rate 3.5 %; 2015/16 UK pounds; UK NHS and Personal Social Services perspective	Women with early and locally advanced breast cancer	• ZA versus no treatment • Risedronate versus no treatment • Clodronate versus no treatment	• ZA versus no treatment: £4974 • Risedronate versus no treatment: – £5045 • Clodronate versus no treatment: £4253	• ZA versus no treatment: 0.09 • Risedronate versus no treatment: 0.76 • Clodronate versus no treatment: 0.23	• ZA versus no treatment: £53,207/QALY • Risedronate versus no treatment: dominant • Clodronate versus no treatment: £18,837/QALY	£20,000/QALY
Huang 2023 [45]	Cost-effectiveness analysis Markov model; discount rate 3 %; 2020 US dollars (6.99 Chinese yuan = 1.00 US dollar); Chinese healthcare provider perspective	Postmenopausal women with HR–positive early breast cancer	ZA plus AI versus AI	$12,247.36	1.09	$11,140.75/QALY	$30,425/QALY

AI, aromatase inhibitor; ET, endocrine therapy; HR, hormone receptor; ICER, incremental cost-effectiveness ratio; ICUR, incremental cost-utility ratio; NA, not applicable; NHS, National Health Service; QALY, quality-adjusted life year; ZA, zoledronic acid.

### 3.1 Zoledronic acid

This systematic review identified 10 RCTs assessing zoledronic acid (ZA) as postoperative adjuvant therapy for EBC patients. The trials comprised six comparing ZA to no treatment, three examining immediate versus delayed ZA administration, and one evaluating five-year versus two-year ZA treatment duration.

#### 3.1.1 ZA vs no treatment

Six RCTs evaluated the efficacy of adjuvant ZA therapy with varying administration schedules. In the two trials, AZURE (BIG 01/04) and NaTaN administered a dose-intensive ZA regimen: 4 mg intravenously every four weeks for six months, then every three months for two years, and every six months for the last 2.5 years for a total of 5 years [17,18,23]. AZURE, the largest study compared ZA with no treatment, enrolled 3359 EBC patients and found that ZA reduced bone metastasis incidence by 24 % (HR 0.76, 95 % CI 0.63–0.92; p = 0.005) yet did not improve disease recurrence, DFS, or OS. A subgroup analysis showed a DFS and invasive DFS benefit for postmenopausal women with ZA, which was not observed in premenopausal or perimenopausal women [17,18].

In contrast, the NaTaN study included 693 high-risk breast cancer patients who did not achieve a pathological complete response following neoadjuvant anthracycline-paclitaxel chemotherapy. After a median follow-up of 54.7 months, no significant differences were observed between the ZA and observation groups regarding bone metastasis-free survival, DFS, and OS. Menopausal status did not significantly influence outcomes in this study [23]. Notely, The NaTaN study's enrollment up to three years post-surgery may have introduced immortal time bias, potentially explaining the observed discrepancies with other adjuvant bisphosphonate studies.

The ABCSG-12 trial, involving 1803 premenopausal estrogen receptor (ER)-positive EBC patients, offered further insights. Participants received goserelin plus tamoxifen or anastrozole and were randomized to intravenous ZA 4 mg every six months for three years or no treatment. A median follow-up of 94.4 months showed ZA reduced the risk of disease progression (HR 0.77, 95 % CI 0.60–0.99; p = 0.042) and death (HR 0.66, 95 % CI 0.43–1.02; p = 0.064), with more pronounced benefits in DFS and OS, observed in previous follow-up analyses [20]. The study suggested that ZA combined with goserelin enhanced the efficacy of adjuvant endocrine therapy with sustained long-term benefits. From an endocrine perspective, all patients in the ABCSG-12 study treated with goserelin achieved ovarian function suppression (OFS), resulting in decreased estrogen levels. Moreover, 77.1 % of participants were older than 40, which may have facilitated more effective ovarian suppression, allowing for estrogen levels to remain within the postmenopausal range [20,48]. Thus, patients enrolled in the ABCSG-12 study may have had estrogen levels similar to those of the postmenopausal subgroup in AZURE, suggesting that patients with low estrogen levels may derive anticancer benefits from ZA.

Similar to the study design of the ABCSG-12 trial, the HOBOE study randomly assigned 1065 premenopausal hormone receptor (HR)-positive EBC patients with ovarian function suppressed by triptorelin to receive adjuvant tamoxifen (N = 354), letrozole (N = 356), or ZA combined with letrozole (N = 355). ZA was administered intravenously at 4 mg every six months for five years [21]. Nevertheless, contrary to the results of ABCSG-12 trial, the HOBOE study found no significant differences in DFS and OS between the letrozole and ZA plus letrozole groups after a median follow-up of 64 months [21]. Though the study's comparative power was limited by a shorter follow-up and fewer events than expected, a recent conference abstract reported results with a median follow-up of 8.6 years, further supporting that ZA did not significantly improve DFS (p = 0.38) and OS (p = 0.25) [49]. The conflicting results between the HOBOE and ABCSG-12 studies imply that prolonging ZA treatment to up to five years may not improve the prognosis of premenopausal EBC patients undergoing OFS. Given the HOBOE trial's three-arm design, limited sample size, and lack of direct comparison between three-year and five-year ZA treatments, further large-scale, prospective RCTs are needed for conclusive evidence.

Two other small studies also evaluated the role of adjuvant ZA. The study by Leal et al. [19] enrolled 68 postmenopausal patients with high-risk breast cancer and compared the efficacy of intravenous ZA (4 mg every 3 months for 1 year) with no treatment. The study by Banys et al. [22] compared intravenous ZA (4 mg monthly for 2 years) with no treatment in 86 patients with breast cancer and bone marrow-disseminated tumour cells. Both studies demonstrated no significant benefit of ZA in reducing the risk of disease recurrence or improving survival, but these findings may be limited by the small sample size [19,22].

#### 3.1.2 Immediate-ZA vs delayed-ZA

Three trials investigated the optimal timing of ZA administration. The E-ZO-FAST, Z-FAST, and ZO-FAST trials shared a consistent design, enrolling postmenopausal HR-positive EBC patients who received adjuvant letrozole and comparing immediate ZA at 4 mg every six months for five years with delayed ZA (after fracture or severe bone mineral density loss) [[24], [25], [26], [27]]. In Z-FAST and E-ZO-FAST, immediate and delayed ZA were not statistically different in disease recurrence and DFS (Z-FAST: P = 0.416; E-ZO-FAST: P = 0.1397) [[24], [25], [26]]. However, the larger ZO-FAST study found a significant DFS advantage with immediate ZA, with a reduction in local recurrence (0.9 % vs 2.3 %) and distant recurrence (5.5 % vs 7.7 %) [27].

The trials' design, not prioritizing DFS as a primary endpoint and initiating ZA in the delayed group, may have reduced the ability to detect DFS differences. This limitation was particularly evident in the smaller Z-FAST and E-ZO-FAST trials because of their lower event rates (Z-FAST: 600 participants with 37 relapses; E-ZO-FAST: 527 participants with 29 relapses) compared to ZO-FAST (1065 participants with 96 relapses) [[24], [25], [26], [27]]. Additionally, the ZO-FAST analysis highlighted particularly pronounced DFS and OS benefits for ZA in patients over 60 or more than five years postmenopausal [27]. These findings align with results from the AZURE trial's postmenopausal subgroup and the ABCSG-12 trial, supporting the hypothesis that the anticancer potential of ZA may be best realized in a low-estrogen environment.

#### 3.1.3 Five-year ZA vs two-year ZA

One study investigated the optimal duration and dosing schedule for ZA. The SUCCESS A trial, a phase III study, randomized 2987 high-risk EBC patients to receive ZA for two years (4 mg intravenously every three months) or five years (4 mg intravenously every three months) after adjuvant chemotherapy [28]. At a median of 5 years after starting ZA, the trial found no significant differences in DFS, OS and distant disease-free survival (DDFS) between the 2-year and 5-year arms, irrespective of pre- or postmenopausal status. Bone recurrence-free survival also showed no statistical difference between groups [28]. In short, for high-risk EBC patients undergoing adjuvant chemotherapy, extending ZA treatment to five years offered no additional prognostic benefits, regardless of menopausal status. It should be noted that the trial did not include a placebo group, limiting the assessment of overall adjuvant ZA therapy benefits.

### 3.2 Clodronate

Four RCTs involving 4976 breast cancer patients evaluated the efficacy of adjuvant clodronate. The prospective clinical trial by Diel et al. [30] first demonstrated the potential benefit of oral clodronate. Among 302 EBC patients with tumor cells detected in bone marrow, those receiving daily oral clodronate (1600 mg for 2 years) had significantly improved OS compared with untreated patients at a median follow-up of 103 months (clodronate group mortality: 20.4 % vs. control: 40.7 %, P = 0.049) [30]. The study by Powles et al. [32] further supports this finding. This trial randomized 1069 patients with stage I-III breast cancer to receive either clodronate (1600 mg/day) or placebo for 2 years. Over the 5-year study period, the clodronate group exhibited a 31 % reduction in bone metastasis risk (HR 0.692, 95 % CI 0.484–0.990, P = 0.043) and a 23 % reduction in mortality risk (HR 0.768, 95 % CI 0.591–0.999, P = 0.048) [32]. Subgroup analysis revealed more pronounced benefits in stage II/III patients (41 % reduction in bone metastasis risk [HR 0.592, 95 % CI 0.398–0.882, P = 0.009] and 26 % reduction in mortality risk [HR 0.743, 95 % CI 0.558–0.989, P = 0.041]) [32], suggesting that this high-risk subgroup may be a potential candidate population for adjuvant clodronate therapy (though validation in larger studies is warranted).

However, the larger NSABP B-34 trial (N = 3323) found no significant prognostic improvement with three years of adjuvant clodronate therapy [31]. Notely, approximately 75 % of participants in this trial exhibited negative axillary lymph nodes, who had a better prognosis and lower recurrence rate than the Powles et al. study cohort may explain the difference in results between the two studies. In addition, subgroup analysis of NSABP B-34 revealed that women over 50 experienced improved recurrence-free, bone metastasis-free and non-bone metastasis-free intervals with clodronate [31], suggesting a potential age-related advantage and supporting the efficacy of bisphosphonates in a low-estrogen setting.

Another randomized study of 282 patients with primary node-positive breast cancer indicated that three years of oral clodronate treatment did not reduce the incidence of bone metastases or mortality risk and even negatively impacted non-bone metastases and DFS, particularly in ER-negative patients [29]. These outcomes may be attributed to a significant imbalance in ER status between the treatment and control groups (25 ER-negative cases in the clodronate group vs. 10 in the control group) and a higher proportion of patients receiving clodronate monotherapy alone without concurrent effective anticancer therapy.

### 3.3 Ibandronate

Three clinical trials, including two phase III and one phase II RCT, evaluated the efficacy of ibandronate. The GAIN trial, a pioneering phase III investigation, randomized EBC patients to daily oral ibandronate at 50 mg for two years or to observation only. It reported no significant improvement in DFS or OS yet noted a non-significant trend toward DFS prolongation in patients younger than 40 and older than 60 years [33].

The TEAM-IIB trial, the largest to date in postmenopausal women with ER-positive breast cancer, also evaluated the effects of adjuvant ibandronate 50 mg once daily for three years. The study found no differences between ibandronate and control groups in overall recurrence, bone recurrence, DFS, or OS [34]. Though the TEAM-IIB trial initially indicated a short-term benefit from ibandronate, this advantage was not statistically significant on multivariate analysis and vanished with extended observation, indicating the possibility of chance findings. The phase II BONADIUV trial then evaluated the effect of an osteoporotic dose of ibandronate (150 mg monthly for two years) in postmenopausal HR-positive EBC patients taking aromatase inhibitors. This study also found no significant differences in the number of invasive DFS events and deaths between groups at a median follow-up of 5.3 years [35].

Oral adjuvant therapy with ibandronate, at both the daily 50 mg and monthly 150 mg doses, did not demonstrate significant benefits. However, the BONADIUV trial may have been limited by the scarcity of events and the short follow-up period. Longer-term studies and larger-scale randomized trials are warranted to achieve a clearer assessment of survival outcomes.

### 3.4 Zoledronic acid vs clodronate vs ibandronate

This review also identified one study that explored which bisphosphonates were recommended for adjuvant therapy for breast cancer. The SWOG S0307 study is currently the only RCT directly comparing the efficacy of various bisphosphonates in patients with stage I-III breast cancer [36]. The trial randomly assigned 6097 patients to 3 years of either intravenous zoledronic acid (given monthly for six months, then every three months for 2.5 years; N = 2262), oral clodronate (1600 mg daily; N = 2268), or oral ibandronate (50 mg daily; N = 1567). It found no significant differences were observed in DFS (log-rank p = 0.49), OS (log-rank p = 0.50), or bone as the first recurrence site (log-rank p = 0.93) among treatments, nor did efficacy vary by patient age or tumor type. Grade 3/4 toxicity was also comparable (zoledronic acid 8.8 %, clodronate 8.3 %, and ibandronate 10.5 %) [36]. However, the absence of a placebo or untreated control in the S0307 trial, similar to the SUCCESS A trial, precludes a comprehensive evaluation of the overall benefit of adjuvant bisphosphonate therapy.

### 3.5 Pamidronate

Two trials compared the efficacy of pamidronate in the adjuvant treatment of EBC, suggesting that the intravenous (IV) form may outperform the oral version [37,42]. Kristensen et al.'s Phase III trial randomized 953 node-negative EBC patients to receive 150 mg oral pamidronate or no treatment for four years, showing no significant benefit in bone recurrence and OS [37], which was consistent with the low oral bioavailability of pamidronate and its limited impact on bone biomarker resorption and potential bone metastasis [50]. In contrast, a smaller non-randomized controlled study found that IV pamidronate at 45 mg every two weeks for four doses significantly decreased bone metastasis incidence and extended bone metastasis-free survival in patients with four or more positive lymph nodes [42]. While suggesting potential for IV pamidronate in EBC adjuvant therapy, its efficacy requires confirmation through large-scale randomized controlled trials.

### 3.6 Denosumab

Two RCTs investigated the efficacy of denosumab, an anti-RANK ligand antibody administered subcutaneously, involving 7929 patients [[38], [39], [40], [41],^51^]. The ABCSG-18 trial of 3420 postmenopausal, HR-positive breast cancer patients treated with an aromatase inhibitor showed that denosumab 60 mg every six months improved DFS, OS, and bone metastasis-free survival (BMFS) compared to placebo [38]. Notably, the DFS benefit was mainly due to new primary tumors and unconfirmed distant metastases, not histologically confirmed breast cancer recurrence [39].

In contrast, the larger D-CARE study with 4509 patients, including 65 % HR-positive and human epidermal growth factor receptor-2 (HER-2) negative, found no significant benefit of denosumab on BMFS, DFS, or OS. Patients received denosumab 120 mg subcutaneously (monthly for six months, then every three months for 4.5 years) or placebo for five years. Although the study included premenopausal women, menopausal status did not influence the outcomes [40].

Compared to the ABCSG-18 trial, the D-CARE trial enrolled a higher-risk patient population for breast cancer recurrence and incorporated a booster dose of denosumab. Additionally, despite denosumab reducing the incidence of bone metastases as the first site of recurrence in the D-CARE trial, the effect was potentially diluted by the approximately 40 % of patients who died before the bone metastases [41]. The composite BMFS endpoint, encompassing bone recurrence, other site recurrences, and all-cause mortality, might also have obscured the clinical impact of denosumab. Thus, varied patient characteristics, endocrine therapies, survival event composition, and treatment regimens between the trials may explain the inconsistent outcomes of adding denosumab to conventional therapy for EBC.

### 3.7 Safety and tolerability

Bone modifiers, including bisphosphonates and denosumab, are typically associated with favorable safety and tolerability. Common side effects mainly include flu-like symptoms, gastrointestinal reactions, and hypercalcemia, which are generally mild and manageable with appropriate symptomatic treatment. Approximately one-third of patients may experience transient flu-like symptoms, such as fever, muscle and bone pain, joint pain, and headaches, following the first administration of bisphosphonates, but usually subside within three days [52]. Gastrointestinal reactions, more common in oral therapy recipients, present as nausea, vomiting, abdominal pain, and diarrhea [53]. Hypocalcemia is a potential consequence due to the inhibitory effect bone modifiers exert on osteoclast-mediated bone resorption. Therefore, serum calcium levels should be monitored, and calcium and vitamin D supplementation is recommended during treatment [54].

Severe adverse events, including nephrotoxicity, osteonecrosis of the jaw (ONJ), and atypical femoral fractures, are rare. Approximately 60 % of bisphosphonates are excreted through the kidneys. This renal clearance makes nephrotoxicity a potential concern, carrying a potential risk of nephrotoxicity, particularly with IV administration. Therefore, it is essential to assess renal function before each infusion. Dose adjustments are mandatory for patients with abnormal renal function [55,56], as reflected in clinical trial protocols like the AZURE and HOBOE trials, where creatinine clearance below 60 ml/min required zoledronic acid dose reduction [17,21]. Unlike bisphosphonates, denosumab does not undergo renal metabolism, so renal function decline should trigger an investigation into other potential causes, not an immediate adjustment in denosumab dosing.

The risk of ONJ is associated with the dosage and route administration of bone modifiers. ONJ with oral bisphosphonates is very rare or has not been reported. However, in the AZURE trial, which used intensive IV therapy, the incidence rate reached 1.8 % [17]. In the S0307 trial, IV zoledronic acid had the highest ONJ incidence at 1.26 %, significantly above that of oral ibandronate (0.77 %) and clodronate (0.36 %) (p = 0.003) [36]. Similar trends were observed with denosumab, where no ONJ cases were reported in the ABCSG-18 trial with semiannual denosumab at 60 mg [39], whereas the risk escalated to 5 % in the D-CARE trial with a more intensive dosing schedule [40]. Given that severe oral disease or invasive dental procedures may increase the ONJ risk, performing a comprehensive oral health assessment before initiating bone modifier therapy and suspending treatment for planned dental interventions are recommended.

Atypical femoral fractures (AFF) are rarely reported in bone modifier treatment. A notable increase in AFF was observed only in the D-CARE trial, recording nine cases (0.4 %) [40]. These fractures often occur with minimal or no trauma, and the healing process is slow. Once an AFF occurs, bone modifier therapy should be discontinued immediately.

### 3.8 Economic considerations

Five studies evaluated the economic impact of adjuvant bisphosphonates in early breast cancer [[43], [44], [45], [46], [47]], with no such analysis found for denosumab. Two studies based on the ABCSG-12 trial suggested that adjuvant zoledronic acid for premenopausal HR-positive EBC patients receiving goserelin exhibited a favorable incremental cost-effectiveness ratio from the perspective of the US and German healthcare systems [44,47]. Nevertheless, caution is warranted in interpreting these findings, as they rely on interim rather than long-term data. The final ABCSG-12 analysis revealed that the risk reduction in disease progression was no longer significant (HR 0.77, P = 0.042) at the predefined level (P = 0.025) compared to the interim analysis (HR 0.64, P = 0.01) [20], which may affect the aforementioned economic studies' validity. Moreover, the economic evaluations based on data from a single trial (ABCSG-12) require corroboration with additional data. Thus, the cost-effectiveness of adding zoledronic acid to endocrine therapy for premenopausal HR + EBC women remains a topic open to further discussion.

Three other studies indicated that adjuvant zoledronic acid is a cost-effective option for postmenopausal EBC patients in Canada, the UK, and China [43,45,46]. In Canada, zoledronic acid was cost-effective for both naturally and induced menopausal women [46]. UK analyses showed that zoledronic acid, clodronate, and ibandronate offered better efficacy and lower costs than no treatment for postmenopausal EBC patients, with clodronate being the most cost-effective. However, in the overall population, zoledronic acid was not cost-effective at the UK willingness-to-pay threshold of £20,000/QALY compared to no treatment. Clodronate and risedronate were deemed cost-effective, with risedronate being the most preferred due to its optimal balance of effectiveness and cost. Among lymph node-positive patients, both zoledronic acid and clodronate were cost-effective, with zoledronic acid providing more benefits at a lower cost than clodronate [43]. Nonetheless, the absence of high-quality clinical evidence to elucidate the distinctions between the various bisphosphonates hinders the ability to perform conclusive economic analyses on their relative cost-effectiveness.

### 3.9 Quality assessment

The Cochrane ROB tool was used to assess the risk of bias in 21 RCTs (results detailed in Supplementary Fig. 1–2). Four studies [31,32,[38], [39], [40], [41]] were judged to have a low risk of bias, while the remaining 17 were judged to have a high risk of bias.

The primary issues in high-risk studies were related to blinding defects, inadequate randomization, attrition bias, and treatment interference bias: all 17 studies employed an open-label design (including the single-blind BONADIUV study [35]), failing to blind participants and researchers, which increased the risk of performance bias; the Leal 2010 study [19] had significant differences in baseline characteristics between groups, suggesting defects in the randomization process; the Banys 2013 study [22] had an attrition rate of 10.4 % (10 out of 96) without specifying the reasons, potentially introducing attrition bias; the Kristensen 2008 study [37] did not permit participants to receive endocrine therapy, while 17 % of participants in the control group and 13 % in the pamidronate group were estrogen receptor-positive. Since these participants did not receive optimal treatment, this could lead to potential bias.

Using the QHES scale, the quality of five economic studies was evaluated and reported in Supplementary Table 2. Each study achieved a high-quality rating, with scores from 84 to 100 and an average of 94.6. 40 % were unclear about their reason for the analytic perspective selected, 60 % did not involve subgroup analysis, and one study inadequately discussed its limitations, potential biases, and conflicts of interest.

### 3.10 Summarization of current clinical practice guidelines

Based on the critical evidence from the EBCTCG meta-analysis, many international guidelines recommend adjuvant bisphosphonate therapy for women with EBC to reduce recurrence and mortality. Most guidelines provide treatment options, including intravenous zoledronic acid, oral clodronate or ibandronate [7,8,57], with the exception of the UK NICE guideline only endorsing zoledronic acid or clodronate [58]. Here, we summarize some of the guidelines' recommendations (Table 4).

**Table 4:** Summarization of current clinical practice guidelines recommendations for adjuvant bisphosphonate treatment of breast cancer.

Guideline	Recommendations
The National Comprehensive Cancer Network (NCCN) Guideline [57]	Adjuvant bisphosphonate therapy is recommended for consideration in patients with high-risk node-negative or node-positive tumors, while adjuvant denosumab is not recommended.
The American Society of Clinical Oncology-Ontario Health (Cancer Care Ontario) (ASCO-OH [CCO]) Guideline [8]	Adjuvant bisphosphonate therapy should be discussed with all postmenopausal patients (natural or therapy-induced) with primary breast cancer, irrespective of hormone receptor status and human epidermal growth factor receptor 2 status, while adjuvant denosumab is not recommended.
The European Society for Medical Oncology (ESMO) Clinical Practice Guideline [7]	Adjuvant bisphosphonates (i.v. zoledronate or daily oral clodronate or ibandronate) are recommended for postmenopausal women or premenopausal women treated with gonadotropin-releasing hormone (GnRH) analogues with early breast cancer deemed at significant risk for recurrence [I, A], while adjuvant denosumab is not recommended.
The National Institute for Health and Care Excellence (NICE), the United Kingdom, guidelines [58]	• Offer bisphosphonates (zoledronic acid or sodium clodronate) as adjuvant therapy to postmenopausal women with node-positive invasive breast cancer. • Consider bisphosphonates (zoledronic acid or sodium clodronate) as adjuvant therapy for postmenopausal women with node-negative invasive breast cancer and a high risk of recurrence.

## 4 Discussion

This systematic review synthesizes evidence from clinical trials and economic studies on the use of bone modifiers (bisphosphonates and denosumab) as adjuvant therapy in early breast cancer. Previously, the EBCTCG and Cochrane groups conducted meta-analyses on the role of bisphosphonates in adjuvant treatment for early breast cancer, both indicating that bisphosphonates can reduce the risk of recurrence and improve survival outcomes in postmenopausal patients to some extent [6,59]. Based on current clinical evidence, our study further found that it is zoledronic acid and clodronate among bisphosphonates that benefit in a low estrogen environment (natural menopause or menopause induced by ovarian function inhibitors). Regarding the efficacy of denosumab, the ABCSG-18 trial [38,39] demonstrated that the standard bone protection dose regimen (60 mg every 6 months) significantly improved DFS in postmenopausal HR-positive breast cancer patients. However, the D-CARE trial [40,41], which used a higher-intensity dose regimen (120 mg monthly for the first 6 months, then every 3 months for 4.5 years) in high-risk patients, did not observe significant survival benefits. Recent data from the GeparX trial [60] further revealed that adding denosumab (120 mg monthly for 6 cycles) to a neoadjuvant chemotherapy containing taxanes/anthracyclines did not improve the pathological complete response (pCR) rate (denosumab group 41.0 % vs. control group 42.8 %, P = 0.58). Compared to the standard bone protection dose used in the ABCSG-18 trial [38,39], both the D-CARE and GeparX trials [40,41,60] employed intensified dose regimens, with the GeparX trial [60] administering denosumab in the neoadjuvant setting where tumor burden is typically higher. However, these intensified strategies failed to translate into additional benefits. Future studies are needed to further explore the role of denosumab in the adjuvant treatment of breast cancer.

For other bone modifiers, international guidelines such as ESMO and ASCO [7,8] recommend ibandronate in addition to zoledronic acid and clodronate, mainly based on the results of the EBCTCG meta-analysis [6] and the S0307 trial [36]. However, current clinical evidence for ibandronate [[33], [34], [35]]has not demonstrated significant benefits in adjuvant therapy for breast cancer. Moreover, the existing clinical evidence is insufficient to conclusively determine the efficacy of other bisphosphonates, such as pamidronate, risedronate, and alendronate, in the adjuvant therapy of early breast cancer. Based on the above evidence, zoledronic acid and clodronate remain the most reliable bone-targeted adjuvant therapy options currently available.

Intravenous zoledronic acid at 4 mg every six months for three years or every three months for two years, and oral clodronate at 1600 mg daily for two years are the most studied bisphosphonate regimens with clear benefits. However, there has yet to be a consensus on the optimal bisphosphonate class, dosage, schedule, or treatment duration. This review hints that immediate use of bisphosphonates may be preferable to delayed use and that more extended or intense therapy is not automatically better. The S0307 study, the sole RCT directly comparing different bisphosphonates, might not be sufficient to clarify the efficacy differences between different bisphosphonates due to the premature closure of the ibandronate group and lower-than-anticipated event rates [36,61]. Additionally, an ABCSG-12 sub-study found no significant DFS and OS differences between patients receiving ≤6 versus ≥7 zoledronic acid infusions [62]. Of note, the number of events in the trial was limited, and the trial could not detect non-inferiority. We are also aware of a pilot trial assessing a single, one-time dose of adjuvant zoledronic acid against the standard six-month dose, which reports only feasibility results thus far [63]. Given the limited available evidence, more studies directly comparing different bisphosphonates, dosages, schedules, and treatment durations are needed.

Another unresolved issue is determining which postmenopausal patients will benefit from bisphosphonate therapy. Further research is required to identify the subgroups of patients most likely to benefit from bisphosphonate therapy, including exploring biomarkers such as circulating tumour cells and MAF gene amplification. The data from individual trials indicate that the absence of MAF gene amplification might be a valuable predictor of benefit from adjuvant bisphosphonate therapy [64,65]. Further research may also hope to investigate the role of adjuvant bisphosphonates according to the genomic risk assessed using multigene assays. Recent studies have identified that a composite score comprising clinical and genetic risk factors could better stratify risk in HR-positive and HER2-negative breast cancer patients. Considering these scores when evaluating the benefits of adjuvant bisphosphonates may provide deeper insight into who might benefit more from these treatments [66,67]. Upcoming studies should also explore the mechanisms underlying differences in response to treatment in different patient subgroups.

Moreover, well-designed studies to further investigate the potential anticancer effects of denosumab in early breast cancer and to compare it with bisphosphonates may be of great interest to the academic and medical communities. Finally, there is a need to study the possible effects of ovarian suppression and bisphosphonate therapy on bone health and future fertility in young people, along with the potential risks of affecting fetal development and the role of adjuvant bisphosphonate therapy in older patients with breast cancer.

On the other hand, in the context of constrained healthcare resources, balancing the potential benefits against the economic costs of bone modifiers to achieve optimal resource allocation is a pivotal issue for healthcare decision-makers and policymakers. Economic evaluations constitute an invaluable reference for these stakeholders. However, existing studies have concentrated on the efficacy and safety of these therapies, often overlooking a detailed economic impact analysis. Our review of the current economic research shows a focus on the economic assessment of zoledronic acid, suggesting it may offer cost savings and a positive cost-effectiveness ratio [43,45,46]. It is essential to recognize that the outcomes of pharmacoeconomic assessments heavily depend on the underlying clinical data inputs. The clinical data underpinning the current economic analyses carry considerable uncertainty, and there is a lack of economic evaluation for other bone modifiers in early breast cancer adjuvant therapy, as well as comparative studies between different modifiers. This knowledge gap complicates the formulation of comprehensive economic conclusions for this field. Therefore, it is recommended that future economic evaluations should encompass a broader spectrum of patient outcome indicators and be anchored in superior-quality clinical data, providing a more comprehensive foundation for informed decision-making.

This systematic review offers a novel perspective by presenting an up-to-date synthesis of clinical trial data and a thorough analysis of the efficacy of bone modifiers in early breast cancer adjuvant treatment. To our knowledge, it is also the first to provide an in-depth synthesis of the economic aspects of these treatments, highlighting their cost-effectiveness—a key factor for healthcare policy and resource allocation.

While this review offers an extensive synthesis of current literature, several limitations are noteworthy. Firstly, the diversity in study design, patient populations, and treatment regimens across trials may restrict the broader applicability of the findings. Future studies should adopt standardized reporting methodologies and include diverse patient populations to bolster the external validity of results. Secondly, economic studies from different countries may not be universally applicable due to differences in healthcare systems, pricing, financial situations, and approval procedures. A further limitation of this review is the limited number of studies, which constrains the potential for definitive conclusions. It would be beneficial for future research to focus on conducting more high-quality randomized controlled trials, which would help to accumulate further scientific evidence and inform more reliable economic studies.

## CRediT authorship contribution statement

**Wenhua Wu:** Writing – original draft, Data curation, Conceptualization. **Yixiao Zhu:** Writing – original draft, Methodology, Data curation. **Huiting Lin:** Writing – original draft, Data curation, Conceptualization. **Jia Liu:** Writing – original draft, Data curation. **Suyan Liu:** Writing – review & editing, Methodology, Investigation. **Lirong Zhang:** Writing – review & editing, Methodology. **Jiaqin Cai:** Writing – review & editing, Data curation. **Hong Sun:** Writing – review & editing, Data curation. **Xiaoxia Wei:** Writing – review & editing, Data curation, Conceptualization.

## Statement

This is a review article and does not contain any original studies involving human participants or animals performed by the authors.

## Funding

This work was supported by the Natural Science Foundation of Fujian, China [grant number 2021J01397]; the Fujian provincial health technology project [grant number 2022GGA010]; and the Fujian provincial Joint Funding Project of Scientific and Technological Innovation [grant numbers 2023Y9347, 2023Y9343, 2023Y9298]. The funding source had no involvement in study design; in the collection, analysis, or interpretation of data; in the writing of the report; or in the decision to submit the article for publication.

## Declaration of competing interest

The authors declare that they have no known competing financial interests or personal relationships that could have appeared to influence the work reported in this paper.
