# Supplementary material for: Clinical and economic research of bone modifiers as adjuvant therapy for early breast cancer: A systematic literature review
Source: Breast. 2025 Aug 7;83:104551. doi: 10.1016/j.breast.2025.104551 (PMC12359659; doi:10.1016/j.breast.2025.104551)
Supplement: Multimedia component 1 [file mmc1.docx]

**Clinical and Economic Research of Bone Modifiers as Adjuvant Therapy for Early Breast Cancer: A** **Systematic Literature Review**

**Supplementary material**

**Supplementary Figure 1.** Risk of bias graph

**Supplementary Figure 2.** Risk of bias summary

**Supplementary Table 1.** Search strategy

**Supplementary Table 2.** The QHES instrument quality assessment

**Supplementary Figure 1.** Risk of bias graph


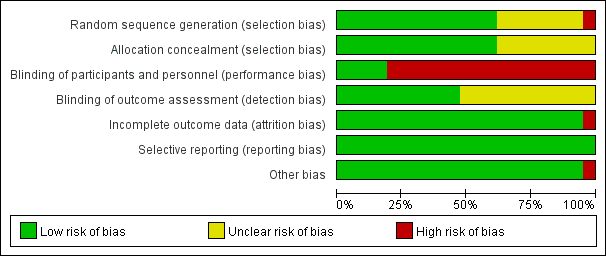


**Supplementary Figure 2.** Risk of bias summary


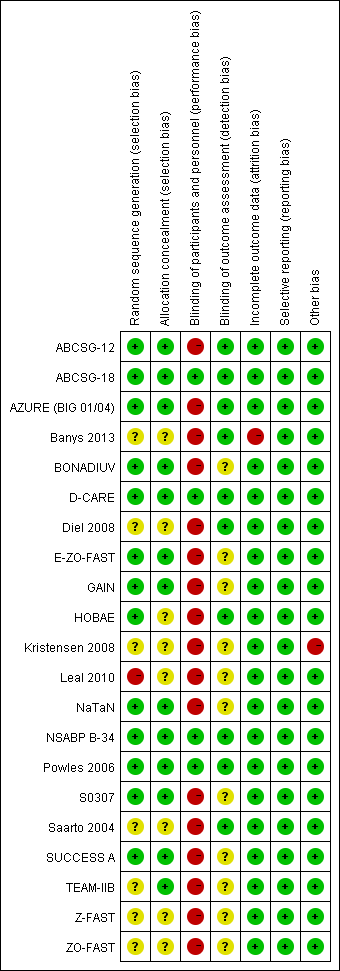


Note: "+" represents a low risk of bias, "?" represents an unclear risk of bias, and "-" represents a high risk of bias.

**Supplementary Table 1.** Search strategy

| **PubMed** | |
| --- | --- |
| #1 | "Diphosphonates"[MeSH Terms] OR "Diphosphonate"[Title/Abstract] OR "bisphosphonate"[Title/Abstract] OR "bisphosphonates"[Title/Abstract] |
| #2 | "Clodronic Acid"[MeSH Terms] OR "acid, clodronic"[Title/Abstract] OR "Dichloromethane Diphosphonate"[Title/Abstract] OR "Dichloromethylenebisphosphonate"[Title/Abstract] OR "Dichloromethylene Biphosphonate"[Title/Abstract] OR "Dichloromethylene Diphosphonate"[Title/Abstract] OR "diphosphonate, dichloromethylene"[Title/Abstract] OR "Cl2MDP"[Title/Abstract] OR "Dichloromethanediphosphonate"[Title/Abstract] OR "Clodronate"[Title/Abstract] OR "Bonefos"[Title/Abstract] |
| #3 | "Etidronic Acid"[MeSH Terms] OR "1-hydroxyethylene diphosphonic acid"[Title/Abstract] OR "Hydroxyethylidene Diphosphonic Acid"[Title/Abstract] OR "Etidronate"[Title/Abstract] OR "1-hydroxyethane-1,1-diphosphonate"[Title/Abstract] OR "1-hydroxyethylidene-1,1-bisphosphonate"[Title/Abstract] OR "EHDP"[Title/Abstract] OR "HEDP"[Title/Abstract] OR "Ethanehydroxydiphosphonate"[Title/Abstract] OR "HEDSPA"[Title/Abstract] OR "Xidifon"[Title/Abstract] OR "Xydiphone"[Title/Abstract] OR "Xidiphon"[Title/Abstract] OR "Didronel"[Title/Abstract] |
| #4 | "Pamidronate"[MeSH Terms] OR "Amino-1-hydroxypropane-1,1-diphosphonate"[Title/Abstract] OR "AHPrBP"[Title/Abstract] OR "Aminopropanehydroxydiphosphonate"[Title/Abstract] OR "Amidronate"[Title/Abstract] OR "Aminohydroxypropylidene Diphosphonate"[Title/Abstract] OR "Pamidronic Acid"[Title/Abstract] OR "Pamidronate Calcium"[Title/Abstract] OR "Pamidronate Disodium"[Title/Abstract] OR "Aredia"[Title/Abstract] |
| #5 | "Alendronate"[MeSH Terms] OR "Aminohydroxybutane Bisphosphonate"[Title/Abstract] OR "MK-217"[Title/Abstract] OR "Alendronate Sodium"[Title/Abstract] OR "Fosamax"[Title/Abstract] |
| #6 | "Ibandronic Acid"[MeSH Terms] OR "Ibandronate"[Title/Abstract] OR "1-Hydroxy-3-(methylpentylamino)propylidenebisphosphonate"[Title/Abstract] OR "Boniva"[Title/Abstract] OR "Bonviva"[Title/Abstract] OR "RPR 102289A"[Title/Abstract] OR "Bondronat"[Title/Abstract] OR "BM 210955"[Title/Abstract] OR "BM 21.0955"[Title/Abstract] |
| #7 | "Zoledronic Acid"[MeSH Terms] OR "CGP 42446A"[Title/Abstract] OR "CGP 42446"[Title/Abstract] OR "Zometa"[Title/Abstract] OR "Zoledronate"[Title/Abstract] |
| #8 | "Risedronic Acid"[MeSH Terms] OR "Atelvia"[Title/Abstract] OR "Actonel"[Title/Abstract] OR "Risedronate"[Title/Abstract] |
| #9 | "YM 529"[Supplementary Concept] OR "YM529"[Title/Abstract] OR "ONO-5920"[Title/Abstract] OR "YH529"[Title/Abstract] OR "minodronate"[Title/Abstract] OR "minodronic acid hydrate"[Title/Abstract] |
| #10 | "Denosumab"[MeSH Terms] OR "Xgeva"[Title/Abstract] OR "AMG 162"[Title/Abstract] OR "Prolia"[Title/Abstract] |
| #11 | #1 OR #2 OR #3 OR #4 OR #5 OR #6 OR #7 OR #8 OR #9 OR #10 |
| #12 | "Breast Neoplasms"[MeSH Terms] OR "Breast Neoplasm"[Title/Abstract] OR "Breast Tumors"[Title/Abstract] OR "Breast Tumor"[Title/Abstract] OR "Breast Cancer"[Title/Abstract] OR "Mammary Cancer"[Title/Abstract] OR "Mammary Cancers"[Title/Abstract] OR "Malignant Neoplasm of Breast"[Title/Abstract] OR "Breast Malignant Neoplasm"[Title/Abstract] OR "Breast Malignant Neoplasms"[Title/Abstract] OR "Malignant Tumor of Breast"[Title/Abstract] OR "Breast Malignant Tumor"[Title/Abstract] OR "Breast Malignant Tumors"[Title/Abstract] OR "Cancer of Breast"[Title/Abstract] OR "Cancer of the Breast"[Title/Abstract] OR "Human Mammary Carcinomas"[Title/Abstract] OR "Human Mammary Carcinoma"[Title/Abstract] OR "Human Mammary Neoplasms"[Title/Abstract] OR "Breast Carcinoma"[Title/Abstract] OR "Breast Carcinomas"[Title/Abstract] |
| #13 | "Randomized Controlled Trials as Topic"[MeSH Terms] OR "randomized controlled trial"[Publication Type] OR "randomized controlled trial"[Title/Abstract] OR "clinical trials as topic"[MeSH Terms] OR "clinical trial"[Publication Type] OR "clinical trials"[Title/Abstract] |
| #14 | "cost benefit"[Title/Abstract] OR "cost minimization"[Title/Abstract] OR "marginal analysis"[Title/Abstract] OR "health technology assessment"[Title/Abstract] OR "Cost-effectiveness"[Title/Abstract] OR "Cost-utility"[Title/Abstract] OR "economic evaluation"[Title/Abstract] OR "Pharmacoeconomics"[Title/Abstract] OR "Budget"[Title/Abstract] OR "Cost"[Title/Abstract] |
| #15 | #13 OR #14 |
| #16 | #11 AND #12 AND #15 |
| **Embase** | |
| #1 | 'bisphosphonic acid derivative'/exp OR ('biphosphonate' OR 'biphosphonates' OR 'bisphosphonate' OR 'bisphosphonates' OR 'diphosphonate derivative' OR 'diphosphonate series' OR 'diphosphonates' OR 'diphosphonic acid derivative' OR 'bisphosphonic acid derivative'):ti,ab,kw |
| #2 | 'clodronic acid'/exp OR ('bm 06011' OR 'bm 6011' OR 'bm06011' OR 'bm6011' OR 'bonefos' OR 'clasteon' OR 'clastoban' OR 'clodron' OR 'clodronate' OR 'clodronato' OR 'clody' OR 'dichloromethanediphosphonate' OR 'dichloromethanediphosphonic acid' OR 'dichloromethylbisphosphonate' OR 'dichloromethylene bisphosphonate' OR 'dichloromethylene diphosphonate' OR 'dichloromethylene diphosphonic acid' OR 'dichloromethylenebisphosphonate' OR 'dichloromethylenebisphosphonic acid' OR 'dichloromethylenediphosphonate' OR 'dichloromethylenediphosphonic acid' OR 'dichloromethylenephosphonate' OR 'difosfonal' OR 'disodium dichloromethane diphosphonate' OR 'disodium dichloromethylidenediphosphonate' OR 'lodronat' OR 'lodronate' OR 'loron' OR 'lytos' OR 'mebonat' OR 'moticlod' OR 'neogrand' OR 'niklod' OR 'ossiten' OR 'ostac' OR 'osteonorm' OR 'sindronat' OR 'traxovical' OR 'clodronic acid'):ti,ab,kw |
| #3 | 'etidronic acid'/exp OR ('1 hydroxyethane 1, 1 diphosphonate' OR '1 hydroxyethane 1, 1 diphosphonic acid' OR '1 hydroxyethyl 1, 1 diphosphonate' OR '1 hydroxyethylene 1, 1 diphosphonate' OR '1 hydroxyethylidene 1, 1 bisphosphonate' OR '1 hydroxyethylidene 1, 1 diphosphonate' OR '1 hydroxyethylidene 1, 1 diphosphonic acid' OR '1 hydroxyethylidenediphosphonate' OR '1 hydroxyethylidenediphosphonic acid' OR 'dequest' OR 'diadronel' OR 'didrocal' OR 'didronal' OR 'didronat' OR 'didronate' OR 'didronel' OR 'difosfen' OR 'dinol (etidronic acid)' OR 'diphos' OR 'diphosphonate' OR 'disodium ethane 1 hydroxyldiphosphonate' OR 'disodium ethanehydroxydiphosphonate' OR 'dronate-os' OR 'editronic acid' OR 'ehdp' OR 'ethane 1 hydroxy 1, 1 bisphosphonate' OR 'ethane 1 hydroxy 1, 1 diphosphonate' OR 'ethane 1 hydroxy 1, 1 diphosphonic acid' OR 'ethane hydroxy diphosphonate' OR 'ethanehydroxy diphosphonate' OR 'ethanehydroxydiphosphonate disodium' OR 'ethylenehydroxydiphosphonate' OR 'etibon' OR 'etidron' OR 'etidronate' OR 'hedp' OR 'hydroxyethylenediphosphonate' OR 'hydroxyethylenediphosphonic acid' OR 'hydroxyethylidenediphosphonic acid' OR 'osteotop' OR 'osteum' OR 'ostopor' OR 'turpinal' OR 'xidiphone' OR 'xydiphone' OR 'xydiphonum' OR 'etidronic acid'):ti,ab,kw |
| #4 | 'pamidronic acid'/exp OR ('1 hydroxy 3 aminopropylidene 1, 1 bisphosphonate' OR '3 amino 1 hydroxy 1, 1 propanediphosphonic acid' OR '3 amino 1 hydroxypropane 1, 1 diphosphonate' OR '3 amino 1 hydroxypropane 1, 1 diphosphonic acid' OR '3 amino 1 hydroxypropylidene 1, 1 bisphosphonate' OR '3 amino 1 hydroxypropylidene 1, 1 bisphosphonic acid' OR '3 amino 1 hydroxypropylidene 1, 1 diphosphonate' OR '3 amino 1 hydroxypropylidene 1, 1 diphosphonic acid' OR '3 amino 1 hydroxypropylidine 1, 1 diphosphonate' OR '3 amino 1, 1 diphosphonopropanol' OR '3 hydroxy 3, 3 diphosphonopropylamine' OR 'amidronate' OR 'aminohydroxypropanediphosphonic acid' OR 'aminohydroxypropyldiphosphonate' OR 'aminohydroxypropylidene 1, 1 diphosphonate' OR 'aminohydroxypropylidene diphosphonate' OR 'aminohydroxypropylidenebisphonic acid' OR 'aminohydroxypropylidenebisphosphonate' OR 'aminomux' OR 'apd' OR 'aredia' OR 'aredronet' OR 'cgp 23339' OR 'ostepam' OR 'pamidran' OR 'pamidrin' OR 'pamidro cell' OR 'pamidromyl' OR 'pamidronat' OR 'pamidronate' OR 'pamidronato' OR 'pamifos' OR 'pamimed' OR 'paminject' OR 'pamipro' OR 'pamired' OR 'pamisol' OR 'pamitor' OR 'panolin' OR 'panorin' OR 'ribodronat' OR 'texpami' OR 'pamidronic acid'):ti,ab,kw |
| #5 | 'alendronic acid'/exp OR ('4 amino 1 hydroxy 1, 1 butanebisphosphonic acid' OR '4 amino 1 hydroxy 1, 1 butanediphosphonic acid' OR '4 amino 1 hydroxybutane 1, 1 diphosphonate' OR '4 amino 1 hydroxybutane 1, 1 diphosphonic acid' OR '4 amino 1 hydroxybutylidene 1, 1 bisphosphonate' OR '4 amino 1 hydroxybutylidene 1, 1 bisphosphonic acid' OR '4 amino 1 hydroxybutylidene 1, 1 diphosphonate' OR '4 amino 1 hydroxybutylidene 1, 1 diphosphonic acid' OR 'adronat' OR 'alenato' OR 'alend' OR 'alendronate' OR 'alendros' OR 'alovell' OR 'arendal' OR 'bifemelan' OR 'bifosa' OR 'binosto' OR 'bonalon' OR 'bonapex' OR 'bonasol' OR 'defixal' OR 'dronal' OR 'endronax' OR 'eucalen' OR 'ex 101' OR 'fixopan' OR 'fosalan' OR 'fosamac' OR 'fosamax' OR 'fosmin' OR 'fosval' OR 'g704650' OR 'genalen' OR 'gth 42' OR 'l 670452' OR 'labr 312' OR 'marvil' OR 'maxibone' OR 'mk 0217' OR 'mk 217' OR 'neobon' OR 'oncalst' OR 'onclast' OR 'osdron' OR 'osdronat' OR 'oseotenk' OR 'osficar' OR 'oslene' OR 'osteofar' OR 'osteofos' OR 'osteopor' OR 'osteosan' OR 'osteovan' OR 'osticalcin' OR 'porosal' OR 'steovess' OR 'teiroc' OR 'tibolene' OR 'voroste' OR 'alendronic acid'):ti,ab,kw |
| #6 | 'ibandronic acid'/exp OR ('1 hydroxy 3 (methyl (pentyl) amino) propane 1, 1 diyldiphosphonic acid' OR '1 hydroxy 3 (methyl) (pentyl) amino 1, 1 propanebisphosphonic acid' OR '1 hydroxy 3 methylpentylaminopropylidene 1, 1 bisphosphonic acid' OR '[1 hydroxy 3 [methyl (pentyl) amino] 1 phosphonopropyl] phosphonic acid' OR 'abrion' OR 'adromux' OR 'adronil' OR 'ascendra (drug)' OR 'axibal' OR 'baxogar' OR 'bm 210955' OR 'bm210955' OR 'bondenza' OR 'bondronat' OR 'bondronate' OR 'bonefurbit' OR 'bonessa' OR 'boniva' OR 'bonmore' OR 'bonnedra' OR 'bonostra' OR 'bonviva' OR 'clastec' OR 'ct 064' OR 'ct064' OR 'destara' OR 'dronaval' OR 'femorel' OR 'flastin' OR 'gerousia' OR 'holmevis' OR 'iasibon' OR 'ibamax' OR 'ibames' OR 'ibamyl' OR 'ibanate' OR 'ibandra' OR 'ibandrix' OR 'ibandro' OR 'ibandrocare' OR 'ibandronate' OR 'ibondem' OR 'ibosat' OR 'idena' OR 'ikametin' OR 'kefort' OR 'lbs 101' OR 'lbs101' OR 'licobondrat' OR 'nucodran' OR 'ostea (drug)' OR 'osteonat' OR 'osteoviva' OR 'phacebonate' OR 'quodixor' OR 'r 484' OR 'r484' OR 'ratiban' OR 'rg 484' OR 'rg484' OR 'ro 2005450' OR 'ro2005450' OR 'rpr 102289a' OR 'rpr102289a' OR 'sedropor' OR 'vinodran' OR 'voliran' OR 'ibandronic acid'):ti,ab,kw |
| #7 | 'zoledronic acid'/exp OR ('(1 hydroxy 2 (1h imidazol 1 yl) 1 phosphonoethyl) phosphonic acid' OR '1 hydroxy 2 (1 imidazolyl) 1, 1 ethanebisphosphonic acid' OR '1 hydroxy 2 (1h imidazol 1 yl) ethylidenebisphosphonic acid' OR '1 hydroxy 2 (imidazol 1 yl) ethylidenebisphosphonic acid' OR 'aclasta' OR 'ak 156' OR 'aszora' OR 'axizoledron' OR 'axs 02' OR 'cgp 42446' OR 'clastizol' OR 'mer 101' OR 'nordeloz' OR 'orazol' OR 'osporil' OR 'reclast' OR 't 121' OR 't121' OR 'zol 446' OR 'zoledon' OR 'zoledro-denk' OR 'zoledron' OR 'zoledronate' OR 'zolenat' OR 'zolimetax' OR 'zomera' OR 'zometa' OR 'zomikos' OR 'zoledronic acid'):ti,ab,kw |
| #8 | 'risedronic acid'/exp OR ('1 hydroxy 2 (3 pyridinyl) ethylidene 1, 1 bisphosphonic acid' OR '1 hydroxy 2 (3 pyridyl) 1, 1 ethanebisphosphonic acid' OR '1 hydroxy 2 (3 pyridyl) ethylidene 1, 1 bisphosphonate' OR '1 hydroxy 2 (3 pyridyl) ethylidene 1, 1 bisphosphonic acid' OR '2 (3 pyridinyl) 1 hydroxyethylidene 1, 1 bisphosphonic acid' OR 'acrel' OR 'actonel' OR 'atelvia' OR 'benet' OR 'ne 58019' OR 'ne 58095' OR 'optinate' OR 'ribastamin' OR 'risedronate' OR 'risedronic acid'):ti,ab,kw |
| #9 | 'minodronic acid'/exp OR ('1 hydroxy 2 (imidazo [1, 2 a] pyridin 3 yl) 1, 1 ethanebisphosphonic acid' OR '1 hydroxy 2 (imidazo [1, 2 a] pyridin 3 yl) ethane 1, 1 bis (phosphonic acid)' OR '1 hydroxy 2 (imidazo [1, 2 a] pyridin 3 yl) ethylidenebisphosphonic acid' OR 'minodronate' OR 'ono 5920' OR 'ono5920' OR 'onobis' OR 'yh 529' OR 'yh529' OR 'ym 529' OR 'ym529' OR 'minodronic acid'):ti,ab,kw |
| #10 | 'denosumab'/exp OR ('amg 162' OR 'amg162' OR 'amgiva' OR 'avt 03' OR 'dyenix' OR 'eb 1001' OR 'fks 518' OR 'gp 2411' OR 'hs 20090' OR 'jhl 1266' OR 'mb 09' OR 'mb09' OR 'mw 032' OR 'pralia' OR 'prolia' OR 'ql 1206' OR 'ranmark' OR 'tk 006' OR 'tvb 009' OR 'xgeva' OR 'denosumab'):ti,ab,kw |
| #11 | #1 OR #2 OR #3 OR #4 OR #5 OR #6 OR #7 OR #8 OR #9 OR #10 |
| #12 | 'breast tumor'/exp OR ('breast gland tumor' OR 'breast gland tumour' OR 'breast mass' OR 'breast neoplasia' OR 'breast neoplasm' OR 'breast neoplasms' OR 'breast tumorigenesis' OR 'breast tumour' OR 'mamma tumor' OR 'mamma tumour' OR 'mammary gland neoplasia' OR 'mammary gland neoplasm' OR 'mammary gland tumor' OR 'mammary gland tumorigenesis' OR 'mammary gland tumour' OR 'mammary neoplasia' OR 'mammary neoplasm' OR 'mammary neoplasms' OR 'mammary tumor' OR 'mammary tumorigenesis' OR 'mammary tumour' OR 'mass in the breast' OR 'masses in the breast' OR 'neoplasia of the breast' OR 'neoplasm of the breast' OR 'neoplasm of the mammary gland' OR 'neoplastic breast' OR 'neoplastic mammary' OR 'neoplastic mammary gland' OR 'tumor of the breast' OR 'tumor of the female breast' OR 'tumor of the mammary gland' OR 'tumorigenesis of the breast' OR 'tumorigenesis of the mammary gland' OR 'breast tumor'):ti,ab,kw |
| #13 | 'randomized controlled trial (topic)'/exp OR 'randomized controlled trials'/exp OR 'clinical trial'/exp |
| #14 | 'cost benefit'/exp OR 'cost minimization'/exp OR 'marginal analysis'/exp OR 'health technology assessment'/exp OR 'Cost-effectiveness'/exp OR 'Cost-utility'/exp OR 'economic evaluation'/exp OR 'Pharmacoeconomics'/exp OR 'Budget'/exp OR 'Cost'/exp |
| #15 | #13 OR #14 |
| #16 | #11 AND #12 AND #15 |
| **Web of science** | |
| #1 | TS=(Diphosphonates OR Diphosphonate OR bisphosphonate OR bisphosphonates) |
| #2 | TS=("Clodronic Acid" OR "Dichloromethane Diphosphonate" OR Dichloromethylenebisphosphonate OR "Dichloromethanediphosphonic Acid" OR "Dichloromethylene Biphosphonate" OR "Dichloromethylene Diphosphonate" OR Cl2MDP OR Dichloromethanediphosphonate OR Clodronate OR Bonefos) |
| #3 | TS=("Etidronic Acid" OR "(1-hydroxyethylene)diphosphonic acid" OR "Hydroxyethylidene Diphosphonic Acid" OR Etidronate OR "1-Hydroxyethane-1,1-Diphosphonate" OR "1-Hydroxyethylidene-1,1-Bisphosphonate" OR EHDP OR "1,1-hydroxyethylenediphosphonate" OR HEDP OR Hydroxyethanediphosphonate OR Ethanehydroxyphosphate OR Ethanehydroxydiphosphonate OR "Phosphonic acid, (1-hydroxyethylidene)bis-, disodium salt" OR "Disodium 1-Hydroxyethylene Diphosphonate" OR HEDSPA OR Xidifon OR Xydiphone OR Xidiphon OR Didronel OR "(1-hydroxyethylene)diphosphonic acid, Tetrapotassium Salt") |
| #4 | TS=(Pamidronate OR "Amino-1-hydroxypropane-1,1-diphosphonate" OR AHPrBP OR Aminopropanehydroxydiphosphonate OR Amidronate OR "(3-Amino-1-hydroxypropylidene)-1,1-biphosphonate" OR "Aminohydroxypropylidene Diphosphonate" OR "1-Hydroxy-3-aminopropane-1,1-diphosphonic acid" OR "Pamidronic Acid" OR Aredia) |
| #5 | TS=(Alendronate OR "4-Amino-1-Hydroxybutylidene 1,1-Biphosphonate" OR "Aminohydroxybutane Bisphosphonate" OR MK-217 OR Fosamax) |
| #6 | TS=("Ibandronic Acid" OR Ibandronate OR "1-Hydroxy-3-(methylpentylamino)propylidenebisphosphonate" OR Boniva OR Bonviva OR RPR-102289A OR Bondronat OR BM-21.0955 OR BM-210955) |
| #7 | TS=("Zoledronic Acid" OR "2-(Imidazol-1-yl)-1-hydroxyethylidene-1,1-bisphosphonic acid" OR "CGP 42446A" OR CGP-42446 OR "CGP-42'446" OR Zometa OR Zoledronate) |
| #8 | TS=("Risedronic Acid" OR "1-Hydroxy-2-(3-pyridyl)ethylidene diphosphonate" OR Atelvia OR "2-(3-pyridinyl)-1-hydroxyethylidene-bisphosphonate" OR Actonel OR Risedronate) |
| #9 | TS=("YM 529" OR "ONO-5920" OR YH-529 OR "(1-hydroxy-2-(imidazo(1,2-a)-pyridin-3-yl)ethylidene)bisphosphonic acid monohydrate" OR minodronate OR "minodronic acid hydrate" OR "1-hydroxy-2-(imidazo(1,2-a)pyridin-3-yl)ethane-1,1-bisphosphonic acid monohydrate" OR "Phosphonic acid, (1-hydroxy-2-imidazo(1,2-a)pyridin-3-ylethylidene)bis-") |
| #10 | TS=(denosumab OR Xgeva OR "AMG 162" OR Prolia) |
| #11 | #1 OR #2 OR #3 OR #4 OR #5 OR #6 OR #7 OR #8 OR #9 OR #10 |
| #12 | TS=("Breast Neoplasms" OR "Breast Neoplasm" OR "Breast Tumors" OR "Breast Tumor" OR "Breast Cancer" OR "Mammary Cancer" OR "Mammary Cancers" OR "Malignant Neoplasm of Breast" OR "Breast Malignant Neoplasm" OR "Breast Malignant Neoplasms" OR "Malignant Tumor of Breast" OR "Breast Malignant Tumor" OR "Breast Malignant Tumors" OR "Cancer of Breast" OR "Cancer of the Breast" OR "Human Mammary Carcinomas" OR "Human Mammary Carcinoma" OR "Human Mammary Neoplasm" OR "Human Mammary Neoplasms" OR "Breast Carcinoma" OR "Breast Carcinomas") |
| #13 | TS=("randomized controlled trial" OR "clinical trials" OR "clinical trial") |
| #14 | TS=("cost benefit" OR "cost minimization" OR "marginal analysis" OR "health technology assessment" OR "Cost-effectiveness" OR "Cost-utility" OR "economic evaluation" OR "Pharmacoeconomics" OR "Budget" OR "Cost") |
| #15 | #13 OR #14 |
| #16 | #11 AND #12 AND #15 |
| **Cochrane library** | |
| #1 | MeSH descriptor: [Diphosphonates] explode all trees |
| #2 | (Diphosphonate OR bisphosphonate OR bisphosphonates):ti,ab,kw |
| #3 | MeSH descriptor: [Clodronic Acid] explode all trees |
| #4 | ("Dichloromethane Diphosphonate" OR Dichloromethylenebisphosphonate OR "Dichloromethanediphosphonic Acid" OR "Dichloromethylene Biphosphonate" OR "Dichloromethylene Diphosphonate" OR Cl2MDP OR Dichloromethanediphosphonate OR Clodronate OR Bonefos):ti,ab,kw |
| #5 | MeSH descriptor: [Etidronic Acid] explode all trees |
| #6 | ("(1-hydroxyethylene)diphosphonic acid" OR "Hydroxyethylidene Diphosphonic Acid" OR Etidronate OR "1-Hydroxyethane-1,1-Diphosphonate" OR "1-Hydroxyethylidene-1,1-Bisphosphonate" OR EHDP OR "1,1-hydroxyethylenediphosphonate" OR HEDP OR Hydroxyethanediphosphonate OR Ethanehydroxyphosphate OR Ethanehydroxydiphosphonate OR "Phosphonic acid, (1-hydroxyethylidene)bis-, disodium salt" OR "Disodium 1-Hydroxyethylene Diphosphonate" OR HEDSPA OR Xidifon OR Xydiphone OR Xidiphon OR Didronel OR "(1-hydroxyethylene)diphosphonic acid, Tetrapotassium Salt"):ti,ab,kw |
| #7 | MeSH descriptor: [Pamidronate] explode all trees |
| #8 | ("Amino-1-hydroxypropane-1,1-diphosphonate" OR AHPrBP OR Aminopropanehydroxydiphosphonate OR Amidronate OR "(3-Amino-1-hydroxypropylidene)-1,1-biphosphonate" OR "Aminohydroxypropylidene Diphosphonate" OR "1-Hydroxy-3-aminopropane-1,1-diphosphonic acid" OR "Pamidronic Acid" OR Aredia):ti,ab,kw |
| #9 | MeSH descriptor: [Alendronate] explode all trees |
| #10 | ("4-Amino-1-Hydroxybutylidene 1,1-Biphosphonate" OR "Aminohydroxybutane Bisphosphonate" OR MK-217 OR Fosamax):ti,ab,kw |
| #11 | MeSH descriptor: [Ibandronic Acid] explode all trees |
| #12 | (Ibandronate OR "1-Hydroxy-3-(methylpentylamino)propylidenebisphosphonate" OR Boniva OR Bonviva OR RPR-102289A OR Bondronat OR BM-21.0955 OR BM-210955):ti,ab,kw |
| #13 | MeSH descriptor: [Zoledronic Acid] explode all trees |
| #14 | ("2-(Imidazol-1-yl)-1-hydroxyethylidene-1,1-bisphosphonic acid" OR "CGP 42446A" OR CGP-42446 OR "CGP-42'446" OR Zometa OR Zoledronate):ti,ab,kw |
| #15 | MeSH descriptor: [Risedronic Acid] explode all trees |
| #16 | ("1-Hydroxy-2-(3-pyridyl)ethylidene diphosphonate" OR Atelvia OR "2-(3-pyridinyl)-1-hydroxyethylidene-bisphosphonate" OR Actonel OR Risedronate):ti,ab,kw |
| #17 | ("YM 529" OR "ONO-5920" OR YH-529 OR "(1-hydroxy-2-(imidazo(1,2-a)-pyridin-3-yl)ethylidene)bisphosphonic acid monohydrate" OR minodronate OR "minodronic acid hydrate" OR "1-hydroxy-2-(imidazo(1,2-a)pyridin-3-yl)ethane-1,1-bisphosphonic acid monohydrate" OR "Phosphonic acid, (1-hydroxy-2-imidazo(1,2-a)pyridin-3-ylethylidene)bis-"):ti,ab,kw |
| #18 | MeSH descriptor: [Denosumab] explode all trees |
| #19 | (Xgeva OR "AMG 162" OR Prolia):ti,ab,kw |
| #20 | #1 OR #2 OR #3 OR #4 OR #5 OR #6 OR #7 OR #8 OR #9 OR #10 OR #11 OR #12 OR #13 OR #14 OR #15 OR #16 OR #17 OR #18 OR #19 |
| #21 | MeSH descriptor: [Breast Neoplasms] explode all trees |
| #22 | ("Breast Neoplasm" OR "Breast Tumors" OR "Breast Tumor" OR "Breast Cancer" OR "Mammary Cancer" OR "Mammary Cancers" OR "Malignant Neoplasm of Breast" OR "Breast Malignant Neoplasm" OR "Breast Malignant Neoplasms" OR "Malignant Tumor of Breast" OR "Breast Malignant Tumor" OR "Breast Malignant Tumors" OR "Cancer of Breast" OR "Cancer of the Breast" OR "Human Mammary Carcinomas" OR "Human Mammary Carcinoma" OR "Human Mammary Neoplasm" OR "Human Mammary Neoplasms" OR "Breast Carcinoma" OR "Breast Carcinomas"):ti,ab,kw |
| #23 | #21 OR #22 |
| #24 | ("randomized controlled trial" OR "clinical trials" OR "clinical trial"):ti,ab,kw |
|  | ("cost benefit" OR "cost minimization" OR "marginal analysis" OR "health technology assessment" OR "Cost-effectiveness" OR "Cost-utility" OR "economic evaluation" OR "Pharmacoeconomics" OR "Budget" OR "Cost"):ti,ab,kw |
| #25 | #23 OR #24 |
| #26 | #20 AND #23 AND #25 |

**Supplementary Table 2.** The QHES instrument quality assessment

| Item No. | Questions | Points | Study ID | | | | |
| --- | --- | --- | --- | --- | --- | --- | --- |
|  |  |  | Delea et al, 2010 | Lux et al, 2010 | Lamond et al, 2015 | National Guideline Alliance (UK), 2018 | Huang et al, 2023 |
| 1 | Was the study objective presented in a clear, specific, and measurable manner? | 7 | Yes | Yes | Yes | Yes | Yes |
| 2 | Were the perspective of the analysis (societal, third-party payer, etc.) and reasons for its selection stated? | 4 | No | Yes | No | Yes | Yes |
| 3 | were variable estimates used in the analysis from the best available source (ie. randomized control trial - best, Expert opinion-worst)? | 8 | Yes | Yes | Yes | Yes | Yes |
| 4 | If estimates came from a subgroup analysis, were the group prespecified at the beginning of the study? | 1 | No | No | Yes | Yes | No |
| 5 | Was uncertainty handled by: 1) statistical analysis to address random events; 2) sensitivity analysis to cover a range of assumptions? | 9 | Yes | Yes | Yes | Yes | Yes |
| 6 | Was incremental analysis performed between alternatives for resources and costs? | 6 | Yes | Yes | Yes | Yes | Yes |
| 7 | Was the methodology for data abstraction (including value health states and other benefits) stated? | 5 | Yes | Yes | Yes | Yes | Yes |
| 8 | Did the analytic horizon allow time for all relevant and important outcomes? Were benefits and costs that went beyond 1 year discounted 3-5%) and justification given for the discount rate? | 7 | Yes | Yes | Yes | Yes | Yes |
| 9 | Was the measurement of costs appropriate and the methodology for the estimation of quantities and unit costs clearly described? | 8 | Yes | Yes | Yes | Yes | Yes |
| 10 | Were the primary outcome measures for the economic evaluation clearly stated and were the major short term, long term and negative outcomes included? | 6 | Yes | Yes | Yes | Yes | Yes |
| 11 | Were the health outcomes measures/scales valid and reliable? If previously tested valid and reliable measures were not available, was justification given for the measures/scales used? | 7 | Yes | Yes | Yes | Yes | Yes |
| 12 | Were the economic model (including structure), study methods and analysis, and the components of the numerator and denominator displayed in a clear transparent manner? | 8 | Yes | Yes | Yes | Yes | Yes |
| 13 | Were the choice of economic model, main assumptions and limitations of the study stated and justified? | 7 | Yes | Yes | Yes | No | Yes |
| 14 | Did the author(s) explicitly discuss direction and magnitude of potential biases? | 6 | Yes | Yes | Yes | No | Yes |
| 15 | Were the conclusions/recommendations of the study justified and based on the study results? | 8 | Yes | Yes | Yes | Yes | Yes |
| 16 | Was there a statement disclosing the source of funding for the study? | 3 | Yes | Yes | Yes | No | Yes |
|  | Total points^*^ | 100 | 95 | 99 | 96 | 84 | 99 |

QHES, Quality of Health Economic Studies Instrument.

A yes answer receives full points for each question, while a no answer receives zero points.

* 0-24 extremely poor quality, 25-49 poor quality, 50-74 fair quality, 75+ high quality.
